# Social stratification in meaningful work: Occupational class disparities in the United Kingdom

**DOI:** 10.1111/1468-4446.12941

**Published:** 2022-04-22

**Authors:** Mark Williams, Jonny Gifford, Ying Zhou

**Affiliations:** ^1^ School of Business and Management Queen Mary University of London London UK; ^2^ Head Office, Chartered Institute of Personnel and Development London UK; ^3^ Surrey Business School, University of Surrey Guildford UK

**Keywords:** class, job attitudes, job quality, meaningful work, occupations, social stratification

## Abstract

Sociologists have long been interested in the meaning workers derive from their jobs. The issue has garnered increasing academic and policy attention in recent years with the concept of “meaningful work,” yet little is known about how social stratification relates to access to it. This paper addresses this issue by exploring how the meaningfulness of jobs—as rated by their incumbents—is stratified across classes and occupations in a national survey of 14,000 working adults in the United Kingdom. It finds modest differentials between classes, with those in routine and manual occupations reporting the lowest levels of meaningfulness and those in managerial and professional occupations and small employers and own account workers reporting the highest levels. Detailed job attributes (e.g., job complexity and development opportunities) explain much of the differences in meaningfulness between classes and occupations, and much of the overall variance in meaningfulness. The main exception is the specific case of how useful workers perceive their jobs to be for society: A handful of occupations relating to health, social care, and protective services which cut across classes stand out from all other occupations. The paper concludes that the modest stratification between classes and occupations in meaningful work is largely due to disparities in underlying job complexity and development opportunities. The extent to which these aspects of work can be improved, and so meaningfulness, especially in routine and manual occupations, is an open, yet urgent, question.

## 1 INTRODUCTION

Sociologists' interest in the sense of meaning derived from work can be dated back to Marx's notion of alienation. This concern was rekindled in recent years with the emergence of the interdisciplinary field of ‘meaningful work’ (Yeoman et al., 2019). While adopting some of the key theoretical underpinnings from pioneering industrial sociology research, positive and organizational psychology have been the more dominant influences (Bailey et al., 2019, p. 89), while the core sociological concern of social stratification (Goldthorpe et al., 1968; Kalleberg & Griffin, 1978; Kohn, 1976) has been all but lost. The early sociological literature went beyond arguing that work itself shaped meaning attitudes by crucially also noting that as the nature of work was highly stratified by occupation, so too, therefore, was the potential level of meaning that could be derived from it. This paper attempts to investigate whether this stratification angle is both relevant and useful in understanding patterns of meaningfulness in today's labor market.

There are a number of reasons for reinvigorating and empirically exploring a stratification perspective in the current context. First, meaningful work has now gained traction to the point where it has become a policy issue: meaningful work is now routinely included alongside other basic working conditions in national job quality frameworks (Carnegie Trust UK, 2018; CIPD, 2018). Second, and relatedly, in wider public discourse, there is now a widespread belief that there is a long tail of jobs with very low scope for meaningfulness, best encapsulated in the popular concept of “bullshit jobs” (Graeber, 2018). However, little evidence exists on whether this is the case or not, nor where these jobs are concentrated. Third, on a theoretical level, social stratification is often defined in terms of occupations: because they are seldom changed, they have more enduring influences on individuals' working lives than jobs or workplaces, where the majority of recent empirical research on meaningful work has focused. Fourth, and relatedly, it is not clear whether occupational class, a conventional way of conceptualizing stratification in sociology, provides an adequate way to map the possible stratification in meaningful work. Graeber's (2018) influential though unsystematic account in many ways challenged this by highlighting the very low levels of meaningfulness felt by the incumbents of economically advantaged jobs such as hedge fund managers, political consultants, and corporate lawyers.

In this paper, we seek to bring some clarity to these issues by exploring occupation and class disparities in meaningful work using the UK Working Lives Survey (UKWLS) 2018 to 2020, a sample of 14,000 working adults in the United Kingdom. We focus our analysis on overall meaningfulness using an index but we also separately explore the three constituent items. Our analysis centers on disparities according to the National Statistics Socio‐Economic Classification (NS‐SEC) alongside detailed occupational categories, and further, how these disparities are accounted for by an unusually rich set of job attributes.

## 2 SOCIAL STRATIFICATION IN MEANINGFUL JOBS

### 2.1 What is meaningful work?

Earlier sociological research operationalized concepts such as alienation and self‐estrangement (Blauner, 1964; Seeman, 1959) as tapping into perceptions of meaning workers attached to their jobs. In recent years, the study of ‘meaningful work’ has emerged as its own interdisciplinary field with its own Oxford University Press handbook and its own biannual conference. Perhaps reflecting its interdisciplinary origins, there is no agreed definition of meaningful work (Bailey et al., 2019). By far the greatest influences on quantitative research in the field has been positive and organizational psychology (Bailey et al., 2019, p. 89), which see meaningfulness as a eudemonic psychological state emanating from the extent to which doing one's job serves the greater good or as an antecedent to engagement emanating from the job tasks (Bailey et al., 2019, pp. 88–92). We focus on three components of meaningfulness relating to what is referred to in the literature as *work significance*, including both a job's perceived usefulness to the production process and its wider social significance (e.g., Steger et al., 2012); and *work purpose*, that is, the extent to which workers find the purpose of their job personally motivating irrespective of whether it is socially significant. Due to data constraints and the lack of a single agreed construct, we exclude various potential components in some definitions such as flourishing, developing, or expressing full potential (Lips‐Wiersma & Wright, 2012; Steger et al., 2012); coherence or being able to make sense of one's work (Martela & Steger, 2016); craftmanship (Pratt et al., 2013; Sennett, 2008); kinship, belonging or social bonds (Pratt et al., 2013); and vocational calling, moral duty, and sacrifice (Bunderson & Thompson, 2009). Some approaches view meaningful work as comprised of related but distinct components (Pratt et al., 2013), while others view components forming a single cohesive or latent construct (e.g., Steger et al., 2012). In this paper, we take a pragmatic approach by exploring disparities in an overall index but recognize it is composed of separate components to the extent it is possible with the dataset we use.

In their negative forms, the foci on perceived work significance, at both organizational and societal levels, and internalized purpose overlap with Graeber's (2018) popular account of meaningless work, or “bullshit jobs.” From testimonies of several hundred workers in dozens of occupations of phenomena such as “pointless” or inexcusably inefficient tasks, and jobs that contribute little or no social value, Graeber drew out a typology of “bullshit jobs,” but he stopped short of drawing out systematic comparisons across occupations. While the book has been critiqued from a sociological perspective (Thompson & Pitts, 2018) and for lacking empirical support, at least in terms of social significance (Soffia et al., 2021), its view that meaningless jobs were prevalent struck a chord in public consciousness. Empirically, meaningful work has been shown to have instrumental value in that it correlates positively with organizational identification, work motivation, and job performance (Michaelson et al., 2014). Partly informed by such research, meaningful work is now routinely included alongside other basic working conditions in national policy definitions of job quality standards (Carnegie Trust UK, 2018; CIPD, 2018). This adds to the pressing need to understand the disparities in the extent to which different kinds of work can be meaningful, an insight which can influence the effectiveness of certain policy responses to promote it.

### 2.2 Social stratification, job attributes, and job attitudes

With a few exceptions (e.g., Soffia et al., 2021), little research has explored how meaningful work is distributed across classes and occupations. While a variety of occupations have been studied in qualitative research including butchers, charity workers, academics, refuse collectors, stonemasons, nurses, and care workers (Bailey & Madden, 2017; Pavlish et al., 2019; Simpson et al., 2014; Taylor & Roth, 2019), most prior research has focused on intra‐occupation comparisons (Bailey & Madden, 2017).

Sociologists' longstanding interest in job attitudes such as meaningfulness includes its differentiation across the occupational structure, in particular according to job attributes. For instance, Blauner (1964) made significant contributions to the empirical study of alienation, which can be thought of as akin to very low meaningfulness, or meaninglessness. His study of factory workers highlighted the monotony of work and lack of participation opportunities led to feelings of alienation, in a critique to the more Marxian notions of the time that all work was “objectively” alienating. Seeman (1959) popularized the related concept of self‐estrangement, reaching somewhat similar conclusions about the critical importance of work organization, including most recently connecting alienating job attributes to health (Seeman et al., 2020).

In the British context, a seminal contribution came from Goldthorpe et al.'s (1968) *The Affluent Worker* studies of men working at car plants in Luton. Although strictly a study about the *meaning* of work as opposed to the present study's focus of *meaningfulness* of work—that is, on “what work signifies (the type of meaning), rather than the *amount* of significance attached to the work” (Rosso et al., 2010, p. 95)—it nevertheless set the stage for decades of research on the British class structure being rooted in conditions of employment, which in turn conditioned worker attitudes.

An enduring finding in these earlier sociological studies was how job attributes influenced the level of meaning workers obtained from their work—independent of their personal characteristics and circumstances. For instance, in the classic study by Kohn (1976), it was the lack of autonomy, variety, and the simplistic tasks of working‐class jobs which explained alienation, rather than ownership or hierarchical position in the capitalist structure (see more generally Kohn & Schooler, 1983 for a seminal contribution on how stratified job attributes influenced stratification in job and non‐job attitudes). In another early study, Kalleberg and Griffin (1978) found intrinsic job quality completely mediated class differences in job satisfaction.

Some of these insights were mirrored in early organizational psychology accounts which formalized many concepts. One influential theory was the Job Characteristics Model (JCM) (Hackman & Oldham, 1975). Although primarily a theory of work motivation, meaningfulness is a key element. It purports that meaningfulness is shaped by three core job characteristics: task variety (the extent to which the job involves a wide or narrow set of tasks), task identity (the extent to which the job requires performing a whole piece of work with a visible outcome or just fragmented elements), and task significance (the extent to which the job is perceived as worthwhile and beneficial for the organization and people's lives). Together with autonomy and feedback, these five core job characteristics exert a significant influence on individuals' psychological states, including meaningfulness (the others being experienced responsibility for the outcome of the work and knowledge of actual results of the work activities), which in turn give rise to intrinsic motivation, job satisfaction, and high‐quality job performance.

What made sociological contributions distinctive from this and the later meaningful work literature was the focus on social stratification in job attributes and job attitudes, in particular the poor (and changing) conditions of the working classes. This can be seen most clearly in accounts focusing on the changing nature of work and the corresponding and unequal changing meaningfulness of work (Strangleman, 2012). An early account in this vein was Bell's (1956) *Work and Its Discontents* which bemoaned how the quest for efficiency in American factories was making work less meaningful. Several decades later, Sennett's (1998) the *Corrosion of Character* was very influential with its account, mirroring Braverman's (1974) account on “deskilling” and “degradation” of the working classes a quarter of a century earlier, whereby the routine factory work found to be so alienating by earlier industrial sociologists, was being replaced by the even more personally restrictive and less psychologically rewarding low‐wage service economy, with Sennett adding growing job insecurity into the mix as another job attribute lowering meaningfulness potential.

On the flipside to working classes allegedly finding less and less meaning in their work were the expanding managerial and professional classes, who enjoyed greater control over their work, more interesting tasks, with greater scope for self‐direction and self‐expression, and so more meaningful work, as well as superior material conditions (Goldthorpe et al., 1968; Kalleberg & Griffin, 1978; Kohn, 1976). However, later social stratification research largely focused on the material conditions of work and much less on more intrinsic job quality and attitudes. The occupational structure has transformed considerably since, with a simultaneous expansion in managerial and professional jobs and a steady decline in intermediate and routine and manual occupations (Williams et al., 2020). Whether managerial and professional occupations continue to enjoy more meaningful work over routine and manual occupations, or are indeed more “bullshit,” are open questions we seek to clarify.

### 2.3 Social stratification, job attributes, and meaningful work

To our knowledge, only a small handful of studies have charted meaningfulness across occupations. Lips‐Wiersma et al. (2016) surveyed 1282 US workers recruited through a convenience sample. Comparing three broad occupational groupings, it found that white‐collar workers tended to report more meaningful work than pink‐collar or blue‐collar workers in three out of four dimensions studied (“unity with others,” “expressing full potential,” and “serving others”) and evidenced no discernible differences in a fourth dimension of “developing inner self.” These findings ran in direct contrast to their theoretical expectations of blue‐ and pink‐collar workers experiencing greater unity with others, given the less abstract nature of such jobs, and pink‐collar workers experiencing higher levels of serving others, given the generally front‐line nature of such jobs.

In a recent study focused on social significance of work, Dur and Van Lent's (2019) analyzed the 2015 International Social Survey Programme and found 10% of UK respondents disagreed with the statement, “My job is useful to society”; and that by this measure, meaningless work was far less likely in the public sector than the private sector, “particularly for occupations such as fire fighters, police officers, social benefits officials, health workers, and teachers” (Dur & Van Lent, 2019, pp. 5–6). In a similar study, Soffia et al. (2021) analyzed the European Working Conditions Survey and found a steady rise in the proportion of workers reporting the feeling of not doing useful work as one moves down the occupational class hierarchy. For instance, while less than 1% of chief executives, senior officials, and legislators felt their jobs were not useful, the figure rises to 6.1% among customer service clerks and 15.2% among laborers in mining, construction, manufacturing, and transport. In line with these and the earlier industrial sociology studies, we expect:

Hypothesis 1: Meaningful work will be socially stratified according to class position, with incumbents of routine and manual occupations rating their jobs as less meaningful than those in managerial and professional jobs.

In addition, building upon the prior social stratification literature, we wish to explore how the social stratification in meaningfulness is explained by job attributes, considering not only intrinsic characteristics of work but a wider suite of variables also including pay and benefits, terms of employment, work–life balance, employee voice, and health and well‐being (Muñoz de Bustillo et al., 2011). Soffia et al. (2021) found feelings of alienation were largely accounted for by poor management but only examined a limited set of factors related to poor management. Nikolova and Cnossen (2020) found autonomy and relatedness accounted for 4.6 times greater variance than extrinsic job attributes such as income, job insecurity, benefits, and working hours. More generally, organizations may have greater decision latitude in how they organize the jobs that compose occupations than is traditionally recognized in stratification research, as a reinvigorated stream of research based on high‐quality employee‐employer linked datasets shows (Avent‐Holt et al., 2020; Wilmers & Aeppli, 2021). Studying detailed job attributes, then, can help to evaluate the extent to which a stratification perspective is useful for understanding how meaningful work is distributed throughout the labor market.

For this paper, we explore 10 job attributes used in policy definitions of job quality or “Good Work” (Carnegie Trust UK, 2018; CIPD, 2018). These include pay and benefits, contracts, job‐demands‐resources, skills, development, job complexity, work‐life balance, work relationships, voice, and health and wellbeing. Factors similar to these are routinely employed in recent sociological job quality and job attribute studies (e.g., Langsæther & Evans, 2020; Warren & Lyonette, 2020) as well as the pioneering industrial sociology research discussed earlier. We do not have prior expectations as to which of these factors may have stronger mediating roles than others. Part of the reason is that the prior literature has not provided a definitive account on this point, with most studies stressing the general importance of job quality or a few selected job attributes. The present analysis is, therefore, meant to be exploratory in order to inform future research. Nevertheless, we expect:

Hypothesis 2: The social stratification predicted in H1 will be explained by the stratification in job attributes across classes.

## 3 DATA AND ANALYTICAL STRATEGY

### 3.1 Data and measures

Data come from the pooled 2018, 2019, and 2020 UKWLS, collected online drawing on YouGov's panel of over 800,000 UK adults who have agreed to take part in surveys. Participants are randomly selected within quotas derived from ONS data according to: the intersection of gender and full‐ or part‐time work status; the intersection of organization size and sector, and industry. Respondents receive small financial incentives for taking part. To minimize response bias, we apply weights, calculated by YouGov based on response rates in relation to the above characteristics (CIPD, 2020). Even still, the final sample is slightly tilted toward those in white‐collar occupations (NS‐SEC 1 to 3), perhaps reflecting that it is an online survey (Table A‐1 in the Online Appendix). Coming from a non‐probability sample, the data are limited for making reliable generalizations about the wider UK population, but the numbers and spread of cases across NS‐SEC categories are sufficient to assume that the disparities identified across them are reliable. In addition, the pooled data afford the advantage of providing the largest sample of meaningful work indicators in a single country (*N* ≈ 14,000), allowing unusually detailed analyses with a rich set of explanatory factors and controls.

Occupations are coded to the ONS' Standard Occupation Classification 2010 derived from written job titles and job descriptions (ONS, 2010a). Occupational class is defined using the ONS' National Statistics Socio‐Economic Classification (NS‐SEC) which allocates respondents based on their SOC codes and other ancillary information to one of the seven classes in Table 1 (ONS, 2010b). We choose NS‐SEC as it is conceptually clear. Although it is primarily designed to capture the clustering of employment relations and the “life chances” associated with different labor market positions (Rose & Pevalin, 2003, 2005), recent research has demonstrated that the occupational categories delineated by it broadly correspond to inequalities in various elements of extrinsic and intrinsic job attributes too, with managerial and professional jobs having the highest job quality in most aspects (CIPD, 2020; Gallie, 2015). We combine large employers, higher managerial and administrative, and higher professional categories given the sample sizes of these groups.

There is no standardized way to measure meaningful work. In a review, Bailey et al. (2019) found a total of 28 meaningful work scales across 56 studies. In the UKWLS, meaningful work was captured using three items, each of which was responded to on a five‐point Likert scale ranging from “Strongly agree” to “Strongly disagree.” As discussed earlier, work significance can occur at an organizational or a societal level, so two separate items were included on this: first “I have the feeling of doing useful work for my organisation” (employed) or “…for my client(s)” (freelancers or independent contractors); and second “I have the feeling of doing useful work for society.” To measure purpose, we used the item “I am highly motivated by my organization's core purpose” (for employed respondents) or “I am highly motivated by the core purpose of my client(s)” (for freelancers or independent contractors). We averaged responses to these items to form an overall meaningfulness index (Cronbach's alpha = .79). We explore results for the index and the three items separately given the view that organizational and societal significance, as well as purpose, may have different disparity patterns.

Since class categorizations may appear quite coarse (Weeden & Grusky, 2012) for our purpose of mapping disparities in meaningfulness, we also explore more detailed occupational categories. Drawing upon the early industrial sociology and the more psychology‐oriented literature that emphasized various job attributes as key antecedents to meaningful work, we propose that meaningful work will be stratified by class position and occupation largely due to differences in job attributes. To explore how differentials may be explained by job attributes, we draw on the 10 CIPD job quality indices (CIPD, 2020). See Table A‐2 for more details on the scale construction and survey items and Table A‐3 for descriptive statistics on them, both in the Online Appendix.

Finally, we employ an extensive set of control variables. We divide these into two groups, demographics and work controls. The demographics are age (five dummies), female (dummy), non‐white ethnicity (dummy), household size (five dummies), qualifications (three dummies), region (eight dummies), year (three dummies). We introduce these because experienced meaningfulness (like all work attitudes) may be influenced by these non‐work factors (Hodson, 2002). The work controls are tenure categories (four dummies), part‐time (dummy), self‐employed (dummy), workplace union (dummy), workplace size (three dummies), industry (four dummies), and key worker status (dummy).

### 3.2 Analytical strategy

The analysis proceeds in five steps. First, we establish class differentials in meaningfulness using Ordinary Least Squares (OLS) regression on the overall meaningfulness index, with NS‐SEC categories as the only independent variable and with higher managerial and professional occupations (the generally most advantaged class) as the reference category. Second, we then control for the demographics listed above. Third, we then control for the work controls listed above. Given the extent of class divisions in the labor market, we generally expect meaningfulness to be robust to the inclusion of these control variables. Fourth, we then control for the 10 job quality attributes to parse out whether observed class differentials in meaningfulness can be accounted for by them. For the first four steps, we report the means and adjusted means in meaningfulness by NS‐SEC in a series of graphs with 95% confidence intervals and omit the controls to simplify the presentation. The underlying regression tables can be found in the Online Appendix. Fifth, given our interest in job attributes as the potential explanatory mechanism, we then decompose the individual roles of each job quality dimension in accounting for class differentials in meaningfulness. Alongside the class‐level analysis, we also report analysis using three constituent meaningfulness items and by more detailed occupational categories (2‐digit).

## 4 RESULTS

Before exploring class differentials in meaningfulness, we begin the analysis with a preliminary exploration of levels in meaningfulness in whole sample in the first column of Table 2. Respondents are much more likely to report their job is useful to their organization than to society, with three‐in‐four respondents agreeing with the former, but less than half agreeing with the latter. A similar proportion is personally motivated by the organization's purpose as with agreeing their job is useful to society. These general patterns are also reflected in the mean scores of the items.

**TABLE 1:** National Statistics Socio‐Economic Classification (NS‐SEC)

Reduced category labels	NS‐SEC category labels	Largest 4‐digit occupations in UKWLS
1. Managerial and professional occupations (M&P)	1. Higher managerial and professional occupations (including large employers) (HMP)	Programmers and software development professionals (employees); Sales accounts and business development managers (employees); Chartered and certified accountants (employees)
	2. Lower managerial and professional occupations (LMP)	Information technology and telecommunications professionals n.e.c. (employees); Finance and investment analysts and advisers (employees); Business sales executives (employees)
2. Intermediate occupations (I)	3. Intermediate occupations (I)	Other administrative occupations n.e.c. (employees); Financial administrative occupations n.e.c. (employees); customer service occupations n.e.c. (employees)
	4. Small employers and own account workers (SE&OA)	Shopkeepers and proprietors—wholesale and retail (small employers); Graphic designers (small employers); pipe fitters (small employers)
3. Routine and manual occupations (R&M)	5. Lower supervisory and lower technical occupations (LST)	Sales and retail assistants (supervisors); Metal working production and maintenance fitters (supervisors); Electricians and electrical fitters (employees)
	6. Semi‐routine occupations (S‐R)	Sales and retail assistants (employees); Care workers and home carers (employees); Postal workers, mail sorters, messengers and couriers (employees)
	7. Routine occupations (R)	Elementary storage occupations (employees); Taxi and cab drivers and chauffeurs (employees); Van drivers (employees)

### 4.1 Class disparities in meaningfulness

Moving to class differentials, beginning with a high‐level descriptive overview, we combine the seven classes into three as per Table 1 and report the percentage point differences in proportions in the final two columns of Table 2. In general, those in routine and manual occupations are less likely to report that their job is useful to the organization, to society, or that they are motivated by their organization's purpose than those in managerial and professional and intermediate occupations. A similar pattern emerges in class differentials in mean scores across each item and the combined index. In general, gaps with managerial and professional occupations are larger for routine and manual occupations than with intermediate occupations.

**TABLE 2:** Meaningfulness by class

	(1)	(2)	(3)	(4)	(5)	(6)
	Total	M&P	I	R&M	Diff. (2)–(3)	Diff. (2)–(4)
*Useful to the organization (%)*						
Strongly disagree	3.2	2.6	3.4	4.4	0.8*	1.9***
Disagree	6.7	6.1	7.3	7.8	1.2*	1.8**
Neither agree nor disagree	15.5	13.5	17.3	18.9	3.8***	5.4***
Agree	54.9	55.4	55.4	52.7	0.0	−2.7*
Strongly agree	19.8	22.4	16.5	16.2	−5.9***	−6.3***
*Useful to society (%)*						
Strongly disagree	7.8	7.5	8.4	8.0	0.9	0.5
Disagree	19.3	18.9	20.7	19.0	1.8*	0.1
Neither agree nor disagree	26.2	24.2	29.0	28.5	4.8***	4.4***
Agree	33.9	34.5	32.5	33.8	−2.0	−0.7
Strongly agree	12.8	15.0	9.4	10.7	−5.6***	−4.3***
*Personally motivated by organization's purpose (%)*						
Strongly disagree	7.5	6.3	7.6	10.6	1.4**	4.3***
Disagree	15.5	14.6	16.2	17.4	1.6*	2.8***
Neither agree nor disagree	29.7	27.1	31.3	34.8	4.2***	7.7***
Agree	35.3	37.9	34.2	29.4	−3.7***	−8.5***
Strongly agree	12.0	14.1	10.6	7.8	−3.6***	−6.3***
*Mean scores*						
Usefulness to organization	3.81	3.89	3.74	3.68	−0.15***	−0.21***
Usefulness to society	3.25	3.31	3.14	3.20	−0.17***	−0.10***
Organization's purpose	3.29	3.39	3.24	3.07	−0.15***	−0.33***
Meaningfulness index	3.45	3.53	3.37	3.32	−0.16***	−0.21***
*N*	13,587	7689	3088	2810	10,777	10,499

*Notes*: M&P are managerial and professional occupations (higher and lower managerial occupations combined), I intermediate occupations (intermediate occupations and small employer and own account workers combined), R&M are routine and manual occupations (lower supervisory and technical, semi‐routine, and routine occupations combined).

* Statistical significance *p* < .05

** *p* < .01

*** *p* < .001.

**FIGURE 1:**
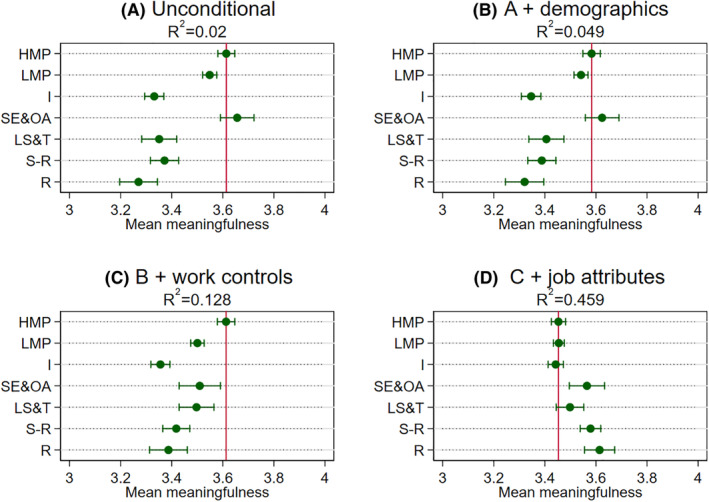
Mean meaningfulness index by class across specifications. Results obtained from OLS models with the overall meaningfulness index as the dependent variable and NS‐SEC categories as the main independent variable (see Table A‐4 in the Online Appendix for underlying models). Dots are means or adjusted means and the horizontal lines are 95% confidence intervals around these estimates. The vertical line is the mean or adjusted mean for higher managerial and professional occupations and can serve as a reference point. HMP are higher managerial and professional occupations, LMP are lower managerial and professional occupations, I are intermediate occupations, SE&OA are small employers and own account workers, LS&T are lower supervisory and technical occupations, S‐R are semi‐routine occupations, and R are routine occupations. See text for controls

**FIGURE 2:**
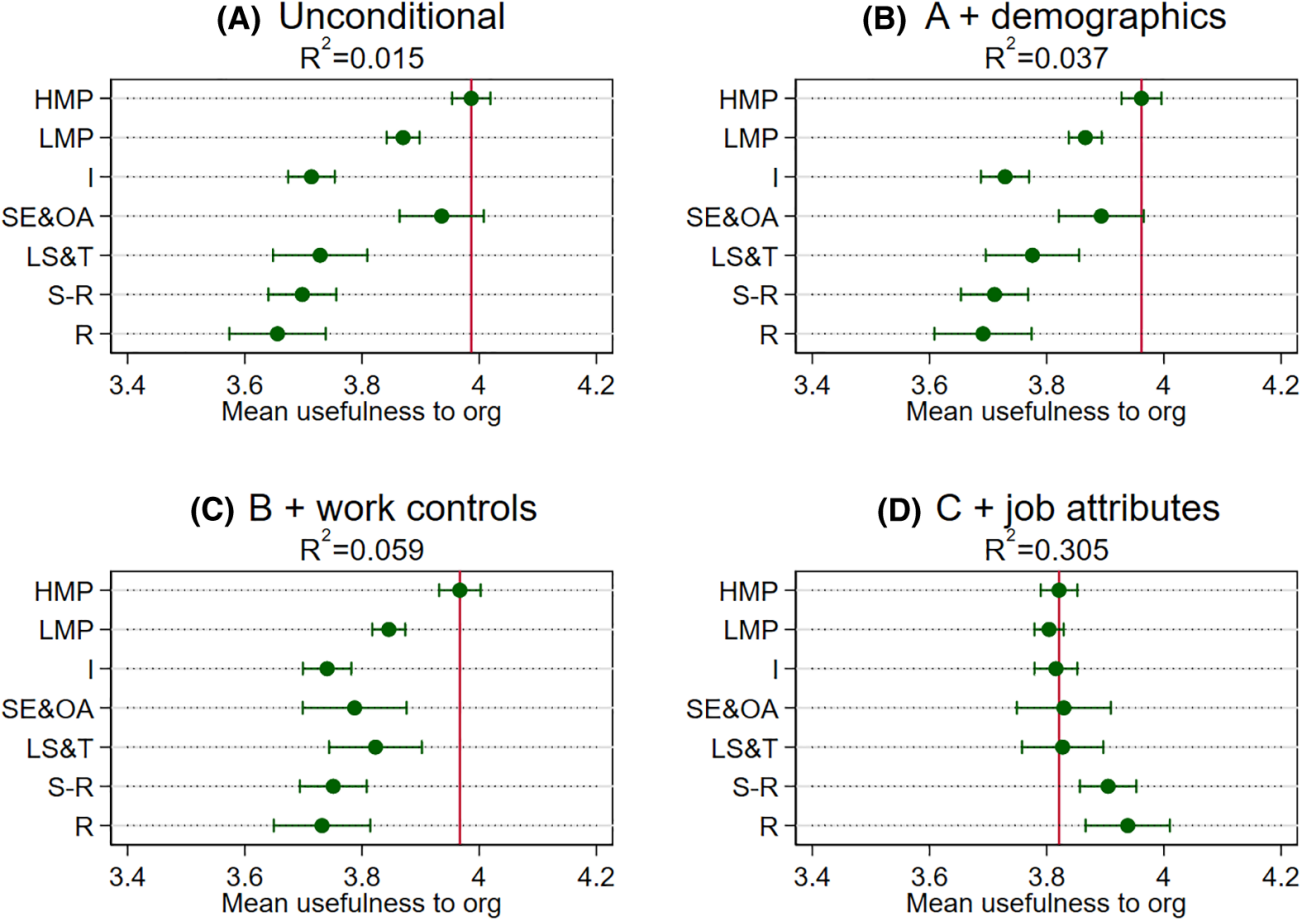
Mean usefulness to the organization by class across specifications. Results obtained from OLS models with usefulness to organization as the dependent variable and NS‐SEC categories as the main independent variable (see Table A‐5 in the Online Appendix for underlying models). Dots are means or adjusted means and the horizontal lines are 95% confidence intervals around these estimates. The vertical line is the mean or adjusted mean for higher managerial and professional occupations and can serve as a reference point. HMP are higher managerial and professional occupations, LMP are lower managerial and professional occupations, I are intermediate occupations, SE&OA are small employers and own account workers, LS&T are lower supervisory and technical occupations, S‐R are semi‐routine occupations, and R are routine occupations. See text for controls

**FIGURE 3:**
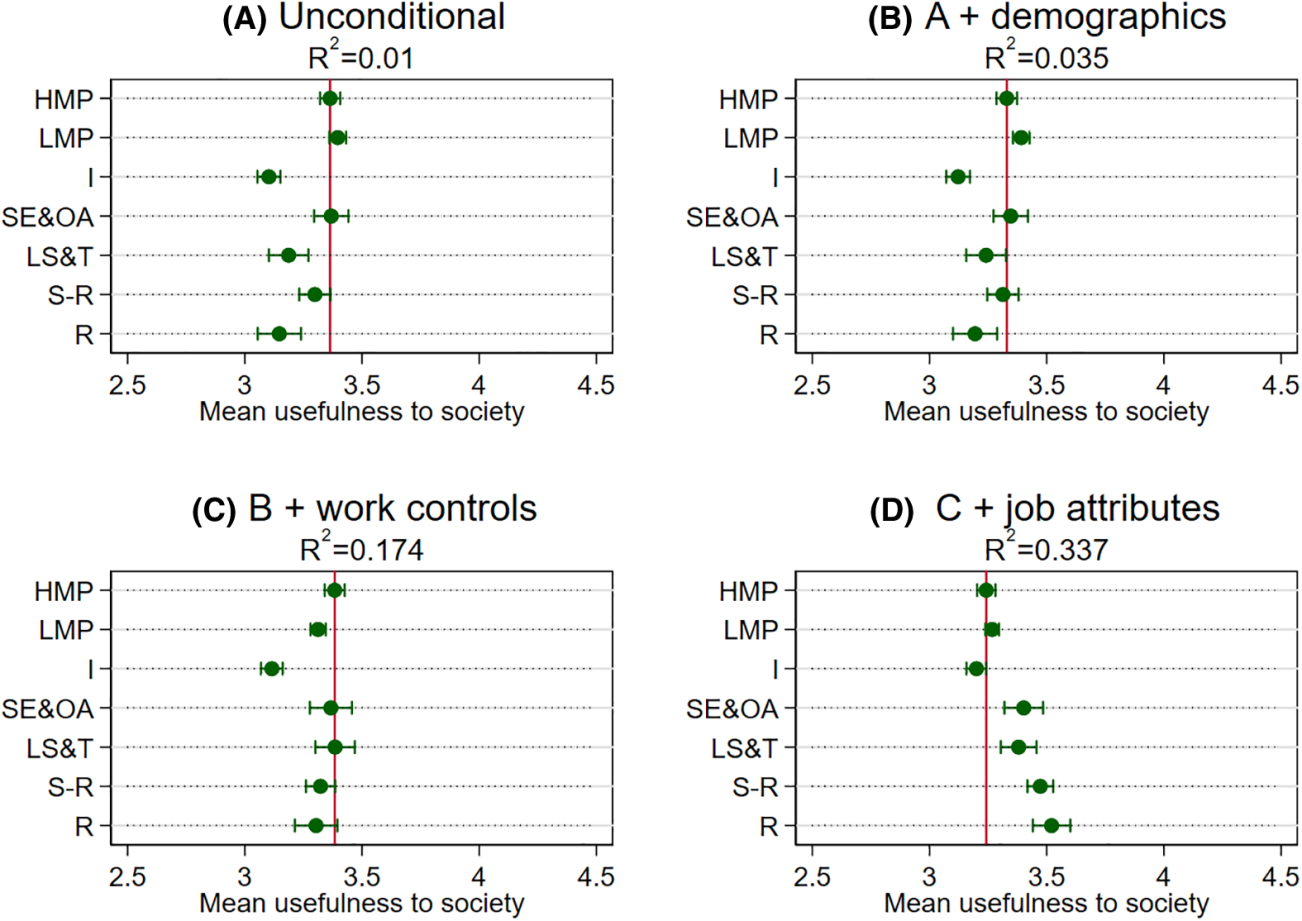
Mean usefulness to society by class across specifications. Results obtained from OLS models with usefulness to society as the dependent variable and NS‐SEC categories as the main independent variable (see Table A‐4 in the Online Appendix for underlying models). Dots are means or adjusted means and the horizontal lines are 95% confidence intervals around these estimates. The vertical line is the mean or adjusted mean for higher managerial and professional occupations and can serve as a reference point. HMP are higher managerial and professional occupations, LMP are lower managerial and professional occupations, I are intermediate occupations, SE&OA are small employers and own account workers, LS&T are lower supervisory and technical occupations, S‐R are semi‐routine occupations, and R are routine occupations. See text for controls

**FIGURE 4:**
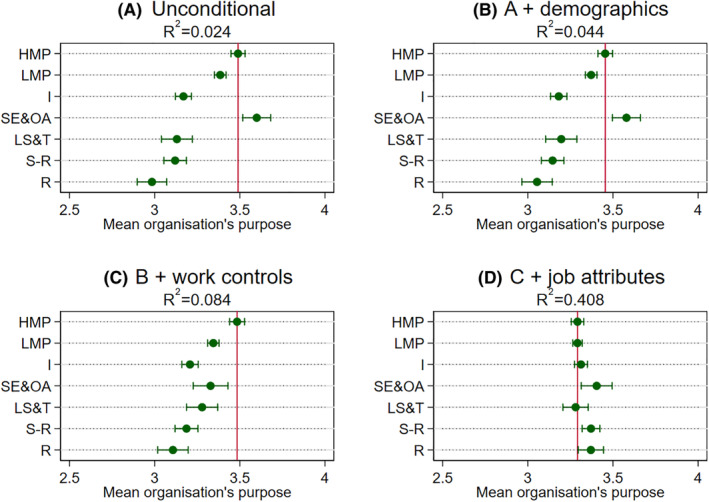
Mean organization's purpose by class across specifications. Results obtained from OLS models with usefulness to society as the dependent variable and NS‐SEC categories as the main independent variable (see Table A‐7 in the Online Appendix for underlying models). Dots are means or adjusted means and the horizontal lines are 95% confidence intervals around these estimates. The vertical line is the mean or adjusted mean for higher managerial and professional occupations and can serve as a reference point. HMP are higher managerial and professional occupations, LMP are lower managerial and professional occupations, I are intermediate occupations, SE&OA are small employers and own account workers, LS&T are lower supervisory and technical occupations, S‐R are semi‐routine occupations, and R are routine occupations. See text for controls

Turning to the multivariable analysis based on the seven‐category NS‐SEC, we next explore whether class disparities might be accounted for by other factors (Figures 1, 2, 3, 4). Panel A in the figures reports the unconditional model (i.e., no controls). As was reported in the descriptive statistics earlier with three “big classes,” we find similar class differentials patterns. The main exception here, though, is in separating out small employers and own account workers from the broader “intermediate class”—this particular group has similar or higher meaningfulness to those in higher managerial and professional jobs. One reason may well be that this class, not being constrained by an employer, enjoy particularly high levels of the intrinsic job characteristics highlighted in the JCM as being central to experienced meaningfulness, even though they are mostly composed of routine and manual occupations such as shop keepers (see Table 1). The job attributes analysis later will shed some light on this.

At this point, it is worth noting that the magnitudes of the coefficients are on the modest side. To get a better handle on magnitudes, we reran the analysis standardizing the four dependent variables to have a mean of 0 and a standard deviation of 1 (reported in Tables 4–7 in the Online Appendix). In general, those in routine and manual jobs report about one‐third of a standard deviation lower meaningfulness than those in higher managerial and professional jobs, which falls to about one‐fifth in the case of usefulness to society. Additionally, the proportion of variance explained (*R*‐squared) by class categories is also modest. Overall, although the modest class differentials found in the Panel As support Hypothesis 1 (that those in higher managerial and professional jobs generally hold an advantageous position), the *R*‐squared quite clearly suggest that other factors are also important in understanding variation in meaningfulness.

When introducing the demographics controls (Panel B in the figures), we find class differentials are not very well explained by demographic factors, although they do add some additional explained variance generally. Regarding our second hypothesis, the general class patterns in the Panel As remain. Introducing the work controls on top of the demographics (Panel Cs), we find the unconditional patterns still largely hold. However, work factors explain some of the apparent similar levels of meaningfulness reported by small employers and own account workers to those in higher managerial and professional jobs in the case of the meaningfulness index, usefulness to the organization, and organization's purpose. Even though class differentials shrink slightly in these specifications, those in higher managerial and professional jobs generally still have the highest adjusted meaningfulness.

The other notable exception is the specific case of usefulness to society. Here, we find the combined demographic and work factors completely explain differentials between the higher managerial and professional and the routine and manual categories. They also account for a larger proportion of the explained variance than is the case for the other meaningfulness indicators. Exploring the more detailed occupation (2‐digit) disparities sheds light on why this is the case (Figure A‐3 in the Online Appendix). It appears there is a handful of occupations that report very high levels of usefulness to society relating to health, social care, and protective services stand out from all other occupations, even when other factors are considered. These in turn cut across the class categories (e.g., including care assistants and doctors, community support officers, and police sergeants). Other research too has identified similar “pro‐social” occupations that do not neatly map onto traditional class groupings (Lambert & Rutherford, 2020). Moreover, they are found in a restricted set of sectors (mostly public sector), which is introduced as a control in the Panel Cs.

### 4.2 The role of job attributes

Including the 10 job attributes in the models on top of demographics and work controls (Panel Ds), we find that class disparities disappear across all four indicators and actually reverse slightly in some instances. In other words, conditional on demographics, work factors, and very detailed job attributes, workers in routine and manual occupations, as well as small employers and own account workers, actually find no different or even slightly greater meaning in their work relative to those in managerial and professional and intermediate jobs, supporting Hypothesis 2. These findings raise further questions about which job attributes are “doing the work” in accounting for class differentials. This specific point is investigated next.

Turning to the mediation decomposition for the overall meaningfulness index (Table 3), which calculates the individual contribution of each job attribute in accounting for the magnitude of the coefficient attached to each class, net of the demographic and work controls, job complexity turns out to be the biggest single factor in explaining the residual meaningfulness gap (i.e., the gap between higher managerial and professional occupations and other classes, net of demographics and work controls). To ease interpretation, these are expressed in percentage terms and the absolute reductions and total gaps explained can be found in Tables A‐8 and A‐9 in the Online Appendix. Job complexity alone accounts for 40% to 50% of the meaningfulness gaps, depending on the class. Job complexity is a summary measure of items relating to the nature of the job tasks such as how monotonous and interesting they are (see Table A‐2 in the Online Appendix). This factor is especially important in explaining the gaps in meaningfulness with those in semi‐routine and routine occupations, accounting for almost all of the gaps.

**TABLE 3:** Decomposition of the mediating role of job quality on occupational class and overall meaningfulness index (% explained)

	LMP	I	SE&OA	LS&T	S‐R	R
Pay and benefits	6.93	4.32	4.46	6.89	4.86	3.27
Contracts	−0.30	0.01	0.93	−1.15	−0.53	−0.50
Demands‐resources	4.27	1.84	1.72	3.52	3.51	2.38
Skills	1.38	1.59	0.98	1.62	1.39	1.36
Development	26.35	29.31	21.55	26.76	20.47	26.33
Job complexity	40.80	50.49	40.54	39.42	52.29	50.67
Work life balance	0.47	−0.61	0.25	−0.43	0.05	−0.15
Relationships	2.75	2.94	4.43	4.93	2.71	2.60
Voice	10.52	10.02	21.24	14.16	10.94	11.95
Health and wellbeing	6.83	0.09	3.91	4.28	4.30	2.09

*Notes*: Decomposition of change in effect size of each class category relative to higher managerial and professional occupations due to the inclusion of each job quality variable conditional on the controls, that is, Model C versus Model D in Figure 1. Expressed in percentage terms. LMP are lower managerial and professional occupations, I are intermediate occupations, SE&OA are small employers and own account workers, LS&T are lower supervisory and technical occupations, S‐R are semi‐routine occupations, and R are routine occupations. See text for controls.

Development is the next most important job attribute, accounting for about one‐quarter of the gaps, depending on the class. This measure is a summary index of items relating to opportunities to develop skills and for career advancement (see Table A‐2 in the Online Appendix). The third most important dimension is voice and representation, accounting for around 10% to 20% of the gap with those in higher managerial and professional occupations. Its role is appreciably larger for small employer and own account workers—jobs with less need for voice given they are generally not embedded in organizations. This aside, voice accounts for about 10% of the meaningfulness gaps between the higher managerial and professional class and the three routine and manual classes. The other seven job attributes play much smaller roles than these three and show no consistent pattern. The findings are broadly replicated when examining their mediating role in the three other items separately (Tables 4, 5, 6).

**TABLE 4:** Decomposition of the mediating role of job quality on occupational class and usefulness to organization (% explained)

	LMP	I	SE&OA	LS&T	S‐R	R
Pay and benefits	8.89	5.68	5.92	8.62	6.17	4.24
Contracts	0.42	−0.02	−1.33	1.56	0.73	0.70
Demands‐resources	6.65	2.93	2.77	5.34	5.39	3.74
Skills	−0.31	−0.37	−0.23	−0.36	−0.31	−0.31
Development	21.47	24.50	18.19	21.28	16.49	21.68
Job complexity	42.25	53.63	43.47	39.83	53.52	53.04
Work life balance	0.51	−0.69	0.28	−0.46	0.05	−0.16
Relationships	4.70	5.15	7.85	8.23	4.58	4.50
Voice	9.30	9.10	19.45	12.22	9.57	10.69
Health and wellbeing	6.12	0.08	3.62	3.75	3.81	1.89

*Notes*: Decomposition of change in effect size of each class category relative to higher managerial and professional occupations due to the inclusion of each job quality variable conditional on the controls, that is, Model C versus Model D in Figure 1. Expressed in percentage terms. LMP are lower managerial and professional occupations, I are intermediate occupations, SE&OA are small employers and own account workers, LS&T are lower supervisory and technical occupations, S‐R are semi‐routine occupations, and R are routine occupations. See text for controls.

**TABLE 5:** Decomposition of the mediating role of job quality on occupational class and usefulness to society (% explained)

	LMP	I	SE&OA	LS&T	S‐R	R
Pay and benefits	−2.00	−1.18	−1.29	−2.02	−1.34	−0.88
Contracts	−0.11	0.00	0.33	−0.42	−0.18	−0.17
Demands‐resources	2.08	0.85	0.84	1.74	1.64	1.09
Skills	4.03	4.40	2.88	4.81	3.90	3.73
Development	28.43	29.96	23.39	29.32	21.12	26.60
Job complexity	49.12	57.59	49.10	48.20	60.21	57.13
Work life balance	0.65	−0.80	0.34	−0.61	0.06	−0.19
Relationships	0.79	0.80	1.28	1.44	0.74	0.70
Voice	9.16	8.28	18.61	12.53	9.12	9.75
Health and wellbeing	7.86	0.10	4.52	5.00	4.73	2.25

*Notes*: Decomposition of change in effect size of each class category relative to higher managerial and professional occupations due to the inclusion of each job quality variable conditional on the controls, that is, Model C versus Model D in Figure 1. Expressed in percentage terms. LMP are lower managerial and professional occupations, I are intermediate occupations, SE&OA are small employers and own account workers, LS&T are lower supervisory and technical occupations, S‐R are semi‐routine occupations, and R are routine occupations. See text for controls.

**TABLE 6:** Decomposition of the mediating role of job quality on occupational class and organization's purpose (% explained)

	LMP	I	SE&OA	LS&T	S‐R	R
Pay and benefits	11.83	7.52	7.38	11.85	8.66	5.81
Contracts	−0.99	0.04	2.93	−3.78	−1.81	−1.69
Demands‐resources	4.05	1.78	1.58	3.36	3.47	2.35
Skills	0.76	0.90	0.53	0.90	0.80	0.79
Development	28.55	32.42	22.67	29.22	23.13	29.72
Job complexity	33.77	42.67	32.57	32.88	45.15	43.70
Work life balance	0.31	−0.41	0.16	−0.29	0.03	−0.10
Relationships	2.68	2.92	4.19	4.84	2.75	2.64
Voice	12.40	12.07	24.30	16.83	13.46	14.68
Health and wellbeing	6.64	0.09	3.68	4.19	4.36	2.11

*Notes*: Decomposition of change in effect size of each class category relative to higher managerial and professional occupations due to the inclusion of each job quality variable conditional on the controls, that is, Model C versus Model D in Figure 1. Expressed in percentage terms. LMP are lower managerial and professional occupations, I are intermediate occupations, SE&OA are small employers and own account workers, LS&T are lower supervisory and technical occupations, S‐R are semi‐routine occupations, and R are routine occupations. See text for controls.

In sum, this exploratory analysis shows that the main reason for the class differentials in meaningfulness—the gaps between managerial and professional occupations and routine and manual occupations—is due to these latter group of occupations having poorer job complexity. Important roles are also found for development prospects and to a lesser extent voice opportunities. This reflects the characteristics highlighted in JCM as being important to explaining experienced meaningfulness. Given these job attributes, which are generally considered as key components of intrinsic job quality, are unevenly distributed across classes, this translates into the class differentials in experienced meaningfulness. Other factors such as the demographic composition of classes and work characteristics (other than job attributes) have a much smaller role. This is also the case in relation to the proportion of overall variance explained, where job attributes add a considerable amount to the R‐squares across all specifications reported. These general findings hold up to whether we consider different demographic subgroups and alternative modeling strategies with respect to the dependent variables (see Tables A‐V in the Online Appendix).

## 5 CONCLUSIONS

Given the issue of meaningful work has garnered increasing attention among social scientists and policy makers in recent years, it is important to understand how it is stratified across positions of advantage and disadvantage emphasized in the sociological literature. Using data from a large national UK survey of adult workers, we found that those in routine and manual jobs find their work less meaningful than those in managerial and professional jobs on average, although the differentials are rather modest. We also found that small employers and own account workers tend to report similar levels of meaningfulness to those in higher managerial and professional occupations, implying a more nuanced class stratification than when it comes to economic advantage. These differentials remain when standard controls such as demographics and work characteristics are taken into account, but disappear (and even reverse) once we control for 10 job attributes. Of the unusually rich set of factors considered, job complexity (how monotonous and interesting the tasks are) stands out as most important. Next important is development opportunities. These findings support the notion that meaningfulness to a great extent depends on intrinsic job quality, which is itself highly stratified by class.

A noteworthy exception to this general pattern is when we considered perceived usefulness to society on its own. In this case, differentials between a small cluster of occupations related to health, social care, and protective services cannot be completely accounted for by the rich set of job attributes we observe. This finding underscores the need to recognize on a theoretical level that social significance needs not coincide with organizational significance or purpose, an important consideration for future research. A practical implication of this finding is that perceived usefulness to society may be more invariant to occupational position. Indeed, socially useful occupations tend to cut across classes.

This paper poses interesting questions on the extent to which organizations and policies can raise levels of meaningfulness within the current social stratification structure. A potentially positive finding in this regard is that classes and occupations themselves explain a small fraction of the overall variance in meaningfulness, whereas job attributes explain a great extent. A potential practical implication is that meaningful work is possible in all occupational classes and interventions to enrich jobs, especially in routine and manual jobs, could benefit employees and organizations. This question is all the more pressing given that mobility across occupations is very low, and if anything, is declining (Williams et al., 2020). The importance of how jobs are designed highlights the role organizations play in shaping and alleviating disparities, and from a theoretical perspective, reinforce the need to go beyond exploring occupational disparities, but to also take into account the role of organizations in how they design the jobs they create. More generally, the fact that job complexity is found to be a key determinant in meaningfulness has wider implications. Long‐term evidence shows that work is generally becoming more routine (Eurofound, 2019; Gallie, 2015). This means that even those in the more privileged labor market positions may have steadily reduced scope for finding meaning in their work. While closing class disparities in job quality should be a key goal, so too should be halting the deterioration in the aspects of intrinsic job quality that enable work to be meaningful.

## Supporting information


Appendix S1

## Data Availability

Data is subject to third party restrictions. The data that support the findings of this study are available from Chartered Institute of Personnel and Development (CIPD). Restrictions apply to the availability of these data, which were used under license for this study. The CIPD can be contacted at https://www.cipd.co.uk/about/contact.
