# Supplementary material for: Social stratification in meaningful work: Occupational class disparities in the United Kingdom
Source: Br J Sociol. 2022 Apr 22;73(3):536–53. doi: 10.1111/1468-4446.12941 (PMC9321196; doi:10.1111/1468-4446.12941)
Supplement: Supplementary file 1 — Appendix S1 [file BJOS-73-536-s001.docx]

**Online Appendix**

Table A-I. NS-SEC distributions in the UK Working Lives Survey (UKWLS) and the Labour Force Survey (LFS)

|  | UKWL 2018 to 2020 (%) | LFS Q12018 to Q12020 (%) | Difference (p.p.) |
| --- | --- | --- | --- |
| NS-SEC: All workers | | | |
| Higher managerial and professional | 19.8 | 17.5 | 2.3 |
| Lower managerial and professional | 32.7 | 28.6 | 4.1 |
| Intermediate | 18.1 | 13.5 | 4.6 |
| Small employers and own account | 8.2 | 11.0 | -2.8 |
| Lower supervisory and technical | 5.3 | 7.5 | -2.2 |
| Semi-routine | 10.4 | 12.7 | -2.3 |
| Routine | 5.4 | 9.2 | -3.8 |
| NS-SEC: Men | | | |
| Higher managerial and professional | 25.4 | 21.4 | 4.0 |
| Lower managerial and professional | 32.7 | 25.2 | 7.5 |
| Intermediate | 11.6 | 7.9 | 3.7 |
| Small employers and own account | 8.3 | 14.1 | -5.8 |
| Lower supervisory and technical | 6.7 | 10.5 | -3.8 |
| Semi-routine | 7.7 | 9.7 | -2.0 |
| Routine | 7.7 | 11.2 | -3.5 |
| NS-SEC: Women | | | |
| Higher managerial and professional | 13.8 | 13.1 | 0.7 |
| Lower managerial and professional | 32.6 | 32.5 | 0.1 |
| Intermediate | 25.2 | 19.9 | 5.3 |
| Small employers and own account | 8.0 | 7.5 | 0.5 |
| Lower supervisory and technical | 3.8 | 4.2 | -0.4 |
| Semi-routine | 13.5 | 16.0 | -2.5 |
| Routine | 3.1 | 6.8 | -3.7 |

Table A-II CIPD Good Work Index (modified)

| Index | Sub-index | | Survey items |
| --- | --- | --- | --- |
| **Pay and benefits** | Subjective pay | | Considering my responsibilities and achievements in my job, I feel I get paid appropriately |
|  | Pension | | Employer pension contribution as a proportion of salary |
|  | Benefits | | Career development benefits in last 12 months |
|  |  |  | Financial assistance benefits in last 12 months |
|  |  |  | Food benefits in last 12 months |
|  |  |  | Health care and insurance benefits in last 12 months |
|  |  |  | Well-being benefits in last 12 months |
|  |  |  | Enhanced leave benefits in last 12 months |
|  |  |  | Social benefits in last 12 months |
|  |  |  | Technology benefits in last 12 months |
|  |  |  | Transport benefits in last 12 months |
| **Contracts** | Security | | How likely to lose job |
|  |  |  | How often work at short notice |
|  |  |  | Permanent in main job |
|  | Underwork | | Hours usually worked per week |
|  |  |  | Hours would like to work per week |
| Job design | **Demand and resources** | Workload | Workload in a normal week |
|  |  | Autonomy | Amount of autonomy in job tasks |
|  |  |  | Amount of autonomy in work pace |
|  |  |  | Amount of autonomy in how work done |
|  |  |  | Amount of autonomy in start or finish time |
|  |  | Resources | I usually have enough time to get my work done within my allocated hours |
|  |  |  | I have the right equipment to do my job effectively |
|  |  |  | I have a suitable space to do my job effectively |
|  | **Skills** | | How well qualified for current job |
|  |  |  | Person-job skills match |
|  | **Development** | | Opportunities to develop skills |
|  |  |  | Prospects for career advancement |
|  | **Job complexity** | | Solving unforeseen problems on your own |
|  |  |  | Monotonous tasks |
|  |  |  | Complex tasks |
|  |  |  | Learning new things |
|  |  |  | Interesting tasks |
| **Work-life balance** | Balance | | I find it difficult to fulfil my commitments outside of work because of the amount of time I spend on my job |
|  |  |  | I find it difficult to do my job properly because of my commitments outside of work |
|  |  |  | I find it difficult to relax in my personal time because of my job |
|  | HR practices | | Taking time out of the day for personal or family matters |
|  |  |  | Flexi-time in last 12 months |
|  |  |  | Job sharing in last 12 months |
|  |  |  | The chance to reduce your working hours in last 12 months |
|  |  |  | Compressed hours in last 12 months |
|  |  |  | Working from home in last 12 months |
|  |  |  | Working only during school term times in last 12 months |
|  | Hours | | Hours usually worked per week including overtime |
|  |  |  | Hours would like to work per week |
|  |  |  | Daily commute time |
| **Relationships at work** | Relationships | | Line manager or supervisor |
|  |  |  | Other managers |
|  |  |  | Colleagues in your team |
|  |  |  | Other colleagues |
|  |  |  | Staff who you manage |
|  |  |  | Customers, clients or service users |
|  |  |  | Suppliers |
|  | Psychological safety | | If I make a mistake, my manager or supervisor will hold it against me |
|  |  |  | People in my team sometimes reject others for being different |
|  |  |  | No one in my team would deliberately act in a way that undermines my efforts |
|  | Line Management | | My boss respects me as a person |
|  |  |  | My boss recognises when I have done a good job |
|  |  |  | My boss is successful in getting people to work together |
|  |  |  | My boss helps me in my job |
|  |  |  | My boss provides useful feedback on my work |
|  |  |  | My boss supports my learning and development |
|  |  |  | My boss can be relied upon to keep their promise |
|  |  |  | My boss is supportive if I have a problem |
|  |  |  | My boss treats me fairly |
| **Voice and representation** | Direct channels | | Employee survey |
|  |  |  | On-line forum or chat room for employees |
|  |  |  | Employee focus groups |
|  |  |  | One-to-one meetings with your line manager |
|  |  |  | Team meetings |
|  |  |  | All department or all organisation meetings |
|  | Indirect channels | | Trade union |
|  |  |  | Non-union staff association or consultation committee |
|  |  |  | How good employee representatives are at seeking the views of employees |
|  |  |  | How good employee representatives are at representing employee views to senior management |
|  |  |  | How good employee representatives are at keeping employees informed of management discussions or decisions |
|  | Management | | How good managers are at seeking the views of employees or employee representatives |
|  |  |  | How good managers are at responding to suggestions from employees or employee representatives |
|  |  |  | How good managers are at allowing employees or employee representatives to influence final decisions |
| **Health and wellbeing** | Physical health | | Impact of work on physical health |
|  |  |  | Backache or other bone, joint or muscle problems (work related, in last year) |
|  |  |  | Breathing problems (work related, in last year) |
|  |  |  | Heart problems (work related, in last year) |
|  |  |  | Hearing problems (work related, in last year) |
|  |  |  | Road traffic accidents while commuting to or from work (work related, in last year) |
|  |  |  | Injury due to an accident while at work (work related, in last year) |
|  |  |  | Repetitive strain injury (RSI) (work related, in last year) |
|  |  |  | Skin problems (work related, in last year) |
|  |  |  | At my work I feel full of energy |
|  |  |  | At my work I feel exhausted |
|  | Mental health | | Impact of work on mental health |
|  |  |  | At my work I feel miserable |
|  |  |  | At my work I feel under excessive pressure |
|  |  |  | Anxiety (work related, in last year) |
|  |  |  | Depression (work related, in last year) |

Notes: Adapted from CIPD (2020). Components in **bold** used in the analysis.

Table A-III. Descriptive statistics of all variables by NS-SEC

|  | NS-SEC | | | | | | |  |
| --- | --- | --- | --- | --- | --- | --- | --- | --- |
|  | HMP | LMP | I | SE&OA | LS&T | S-R | R | Total |
|  |  |  |  |  |  |  |  |  |
| Useful to organisation/client | 3.95 | 3.87 | 3.77 | 4.00 | 3.83 | 3.71 | 3.87 | 3.86 |
| Useful to society | 3.31 | 3.39 | 3.20 | 3.55 | 3.19 | 3.38 | 3.40 | 3.34 |
| Organisation/client’s purpose | 3.43 | 3.29 | 3.12 | 3.56 | 3.20 | 3.23 | 3.23 | 3.29 |
| Meaningful work index | 3.56 | 3.52 | 3.36 | 3.73 | 3.41 | 3.43 | 3.51 | 3.49 |
| Usefulness to organisation/client (standardised) |  |  |  |  |  |  |  |  |
| Usefulness to society (standardised) |  |  |  |  |  |  |  |  |
| Organisation/client’s purpose (standardised) |  |  |  |  |  |  |  |  |
| Meaningful work index (double standardised) |  |  |  |  |  |  |  |  |
| Survey year: 2018 | 0.00 | 0.00 | 0.00 | 0.00 | 0.00 | 0.00 | 0.00 | 0.00 |
| Survey year: 2019 | 0.00 | 0.00 | 0.00 | 0.00 | 0.00 | 0.00 | 0.00 | 0.00 |
| Survey year: 2020 | 1.00 | 1.00 | 1.00 | 1.00 | 1.00 | 1.00 | 1.00 | 1.00 |
| Age: 18-29 | 0.08 | 0.06 | 0.03 | 0.03 | 0.02 | 0.02 | 0.01 | 0.05 |
| Age: 30-39 | 0.22 | 0.18 | 0.14 | 0.06 | 0.14 | 0.07 | 0.08 | 0.16 |
| Age: 40-49 | 0.23 | 0.26 | 0.27 | 0.17 | 0.19 | 0.18 | 0.17 | 0.23 |
| Age: 50-59 | 0.28 | 0.32 | 0.36 | 0.34 | 0.41 | 0.47 | 0.41 | 0.35 |
| Age: 60+ | 0.19 | 0.19 | 0.20 | 0.40 | 0.25 | 0.25 | 0.32 | 0.22 |
| Female | 0.31 | 0.44 | 0.60 | 0.37 | 0.35 | 0.60 | 0.16 | 0.43 |
| Other than white ethnicity | 0.12 | 0.08 | 0.07 | 0.02 | 0.03 | 0.05 | 0.06 | 0.08 |
| Household size: 1 | 0.19 | 0.21 | 0.24 | 0.25 | 0.28 | 0.23 | 0.24 | 0.22 |
| Household size: 2 | 0.42 | 0.38 | 0.41 | 0.40 | 0.40 | 0.45 | 0.44 | 0.41 |
| Household size: 3 | 0.17 | 0.20 | 0.16 | 0.19 | 0.20 | 0.14 | 0.18 | 0.18 |
| Household size: 4 | 0.16 | 0.14 | 0.15 | 0.10 | 0.09 | 0.14 | 0.10 | 0.14 |
| Household size: 5 | 0.05 | 0.06 | 0.03 | 0.05 | 0.03 | 0.06 | 0.05 | 0.05 |
| Qualifications: Below degree | 0.24 | 0.41 | 0.60 | 0.58 | 0.59 | 0.63 | 0.71 | 0.46 |
| Qualifications: Undergraduate | 0.35 | 0.33 | 0.22 | 0.29 | 0.20 | 0.21 | 0.14 | 0.28 |
| Qualifications: Postgraduate | 0.41 | 0.26 | 0.17 | 0.13 | 0.21 | 0.16 | 0.14 | 0.25 |
| Region: Northern England | 0.25 | 0.24 | 0.28 | 0.17 | 0.28 | 0.21 | 0.22 | 0.24 |
| Region: Midlands | 0.15 | 0.16 | 0.17 | 0.18 | 0.12 | 0.14 | 0.26 | 0.16 |
| Region: East England | 0.09 | 0.10 | 0.10 | 0.10 | 0.07 | 0.13 | 0.05 | 0.10 |
| Region: London | 0.15 | 0.14 | 0.11 | 0.08 | 0.06 | 0.08 | 0.14 | 0.12 |
| Region: Southern England | 0.23 | 0.22 | 0.21 | 0.35 | 0.24 | 0.24 | 0.13 | 0.23 |
| Region: Wales | 0.04 | 0.06 | 0.06 | 0.07 | 0.12 | 0.12 | 0.09 | 0.07 |
| Region: Scotland | 0.06 | 0.04 | 0.03 | 0.05 | 0.06 | 0.04 | 0.07 | 0.05 |
| Region: Northern Ireland | 0.03 | 0.05 | 0.03 | 0.00 | 0.04 | 0.03 | 0.03 | 0.03 |
| Tenure: <=1 year | 0.09 | 0.09 | 0.05 | 0.08 | 0.05 | 0.09 | 0.08 | 0.08 |
| Tenure: <=5 years | 0.29 | 0.25 | 0.27 | 0.19 | 0.19 | 0.33 | 0.33 | 0.27 |
| Tenure: <=15 years | 0.35 | 0.36 | 0.36 | 0.40 | 0.50 | 0.37 | 0.44 | 0.37 |
| Tenure: 15+ | 0.27 | 0.30 | 0.32 | 0.33 | 0.26 | 0.21 | 0.14 | 0.28 |
| Part-time | 0.14 | 0.21 | 0.31 | 0.37 | 0.19 | 0.49 | 0.30 | 0.26 |
| Self-employed | 0.06 | 0.10 | 0.01 | 0.93 | 0.02 | 0.01 | 0.00 | 0.10 |
| Temporary contract | 0.02 | 0.01 | 0.01 | 0.00 | 0.02 | 0.01 | 0.05 | 0.01 |
| Union | 0.15 | 0.23 | 0.22 | 0.00 | 0.13 | 0.25 | 0.22 | 0.19 |
| Workplace size: 1-9 workers | 0.15 | 0.20 | 0.15 | 0.99 | 0.11 | 0.13 | 0.13 | 0.21 |
| Workplace size: 10-249 | 0.34 | 0.24 | 0.26 | 0.01 | 0.39 | 0.19 | 0.45 | 0.26 |
| Workplace size: 250+ | 0.51 | 0.56 | 0.60 | 0.01 | 0.50 | 0.68 | 0.42 | 0.53 |
| Industry: Industry | 0.15 | 0.12 | 0.14 | 0.30 | 0.30 | 0.16 | 0.25 | 0.16 |
| Industry: Consumer services | 0.14 | 0.28 | 0.27 | 0.52 | 0.53 | 0.56 | 0.66 | 0.32 |
| Industry: Business services | 0.48 | 0.22 | 0.25 | 0.09 | 0.04 | 0.01 | 0.02 | 0.24 |
| Industry: Public services | 0.23 | 0.39 | 0.34 | 0.09 | 0.13 | 0.27 | 0.08 | 0.28 |
| Work-Life Balance | 0.52 | 0.51 | 0.54 | 0.57 | 0.51 | 0.55 | 0.54 | 0.53 |
| Pay and Benefits | 0.52 | 0.45 | 0.44 | 0.40 | 0.42 | 0.40 | 0.46 | 0.46 |
| Contracts Index | 0.88 | 0.87 | 0.89 | 0.81 | 0.86 | 0.84 | 0.85 | 0.87 |
| Job Complexity | 0.68 | 0.63 | 0.58 | 0.62 | 0.59 | 0.51 | 0.51 | 0.61 |
| Relationships at Work | 0.73 | 0.72 | 0.70 | 0.95 | 0.67 | 0.69 | 0.67 | 0.72 |
| Employee Voice | 0.34 | 0.33 | 0.32 | 0.02 | 0.31 | 0.31 | 0.30 | 0.31 |
| Health and Wellbeing | 0.57 | 0.56 | 0.55 | 0.60 | 0.58 | 0.56 | 0.59 | 0.57 |
| Demand and resources | 0.70 | 0.64 | 0.64 | 0.77 | 0.65 | 0.61 | 0.64 | 0.66 |
| Skills | 0.61 | 0.61 | 0.60 | 0.63 | 0.53 | 0.49 | 0.49 | 0.58 |
| Development | 0.57 | 0.52 | 0.44 | 0.47 | 0.45 | 0.43 | 0.44 | 0.50 |

Notes: HMP are higher managerial and professional occupations, LMP are lower managerial and professional occupations, I are intermediate occupations, SE&OA are small employers and own account workers, LS&T are lower supervisory and technical occupations, S-R are semi-routine occupations, and R are routine occupations.

Table A-IV. Figure I regression results in full (standardised meaningfulness index)

|  | (1) | (2) | (3) | (4) |
| --- | --- | --- | --- | --- |
| Higher managerial and professional (reference) |  |  |  |  |
|  |  |  |  |  |
| Lower managerial and professional | -0.0660^**^ | -0.0436^*^ | -0.109^***^ | 0.000263 |
|  | (0.021) | (0.021) | (0.020) | (0.016) |
| Intermediate | -0.271^***^ | -0.227^***^ | -0.245^***^ | -0.00957 |
|  | (0.024) | (0.026) | (0.025) | (0.020) |
| Small employers and own account | 0.0359 | 0.0337 | -0.104^*^ | 0.101^**^ |
|  | (0.036) | (0.036) | (0.043) | (0.037) |
| Lower supervisory and technical | -0.253^***^ | -0.171^***^ | -0.113^**^ | 0.0410 |
|  | (0.037) | (0.037) | (0.038) | (0.029) |
| Semi-routine | -0.237^***^ | -0.192^***^ | -0.190^***^ | 0.117^***^ |
|  | (0.031) | (0.032) | (0.032) | (0.025) |
| Routine | -0.330^***^ | -0.253^***^ | -0.218^***^ | 0.152^***^ |
|  | (0.040) | (0.041) | (0.041) | (0.033) |
| Survey year: 2018 (reference) |  |  |  |  |
|  |  |  |  |  |
| Survey year: 2019 |  | 0.0631^**^ | 0.0500^**^ | 0.0598^***^ |
|  |  | (0.019) | (0.019) | (0.015) |
| Survey year: 2020 |  | 0.0166 | 0.0101 | 0.0631^***^ |
|  |  | (0.019) | (0.018) | (0.015) |
| Age: 18-29 (reference) |  |  |  |  |
|  |  |  |  |  |
| Age: 30-39 |  | 0.0837^*^ | 0.0738^*^ | 0.0805^**^ |
|  |  | (0.035) | (0.034) | (0.027) |
| Age: 40-49 |  | 0.147^***^ | 0.105^**^ | 0.141^***^ |
|  |  | (0.034) | (0.033) | (0.026) |
| Age: 50-59 |  | 0.150^***^ | 0.0991^**^ | 0.178^***^ |
|  |  | (0.033) | (0.033) | (0.026) |
| Age: 60+ |  | 0.319^***^ | 0.255^***^ | 0.238^***^ |
|  |  | (0.035) | (0.035) | (0.028) |
| Female |  | 0.100^***^ | 0.0433^**^ | 0.0608^***^ |
|  |  | (0.016) | (0.016) | (0.013) |
| Other than white ethnicity |  | 0.0428 | 0.0330 | 0.00114 |
|  |  | (0.032) | (0.032) | (0.025) |
| Household size: 1 |  |  |  |  |
|  |  |  |  |  |
| Household size: 2 |  | 0.0146 | 0.0274 | -0.0142 |
|  |  | (0.021) | (0.021) | (0.017) |
| Household size: 3 |  | -0.0234 | -0.0124 | -0.0506^**^ |
|  |  | (0.025) | (0.024) | (0.019) |
| Household size: 4 |  | 0.0418 | 0.0593^*^ | -0.0190 |
|  |  | (0.027) | (0.026) | (0.020) |
| Household size: 5 |  | 0.0563 | 0.0715 | -0.0220 |
|  |  | (0.038) | (0.037) | (0.029) |
| Qualifications: Below degree |  |  |  |  |
|  |  |  |  |  |
| Qualifications: Undergraduate |  | 0.116^***^ | 0.0717^***^ | 0.0381^*^ |
|  |  | (0.020) | (0.019) | (0.015) |
| Qualifications: Postgraduate |  | 0.253^***^ | 0.178^***^ | 0.0933^***^ |
|  |  | (0.020) | (0.019) | (0.016) |
| Region: Northern England |  |  |  |  |
|  |  |  |  |  |
| Region: Midlands |  | -0.0368 | -0.0254 | 0.000293 |
|  |  | (0.026) | (0.025) | (0.019) |
| Region: East England |  | -0.0538 | -0.0376 | -0.0296 |
|  |  | (0.031) | (0.030) | (0.024) |
| Region: London |  | -0.0229 | 0.0177 | 0.0177 |
|  |  | (0.029) | (0.027) | (0.022) |
| Region: Southern England |  | 0.00518 | 0.0295 | 0.0212 |
|  |  | (0.022) | (0.021) | (0.017) |
| Region: Wales |  | -0.0434 | -0.0397 | -0.0343 |
|  |  | (0.039) | (0.037) | (0.030) |
| Region: Scotland |  | 0.0593^*^ | 0.0744^**^ | 0.0444^*^ |
|  |  | (0.028) | (0.027) | (0.022) |
| Region: Northern Ireland |  | -0.0456 | -0.0347 | -0.0242 |
|  |  | (0.080) | (0.078) | (0.062) |
| Tenure: <=1 year (reference) |  |  |  |  |
|  |  |  |  |  |
| Tenure: <=5 years |  |  | -0.0423 | 0.0186 |
|  |  |  | (0.027) | (0.021) |
| Tenure: <=15 years |  |  | -0.0924^***^ | -0.0338 |
|  |  |  | (0.027) | (0.021) |
| Tenure: 15+ |  |  | -0.0826^**^ | -0.0282 |
|  |  |  | (0.029) | (0.023) |
| Part-time |  |  | -0.00653 | 0.0389^*^ |
|  |  |  | (0.019) | (0.017) |
| Self-employed |  |  | 0.151^***^ | 0.135^***^ |
|  |  |  | (0.033) | (0.030) |
| Temporary contract |  |  | 0.0312 | 0.0197 |
|  |  |  | (0.048) | (0.042) |
| Union |  |  | 0.101^***^ | -0.000134 |
|  |  |  | (0.022) | (0.019) |
| Workplace size: 1-9 workers (reference) |  |  |  |  |
|  |  |  |  |  |
| Workplace size: 10-249 |  |  | -0.0773^**^ | -0.0591^**^ |
|  |  |  | (0.026) | (0.021) |
| Workplace size: 250+ |  |  | -0.159^***^ | -0.157^***^ |
|  |  |  | (0.026) | (0.021) |
| Industry: Industry (reference) |  |  |  |  |
|  |  |  |  |  |
| Industry: Consumer services |  |  | -0.0221 | 0.0635^***^ |
|  |  |  | (0.023) | (0.018) |
| Industry: Business services |  |  | -0.0229 | -0.0525^**^ |
|  |  |  | (0.024) | (0.018) |
| Industry: Public services |  |  | 0.506^***^ | 0.449^***^ |
|  |  |  | (0.025) | (0.020) |
| Work-Life Balance |  |  |  | 0.0848^*^ |
|  |  |  |  | (0.043) |
| Pay and Benefits |  |  |  | 0.152^***^ |
|  |  |  |  | (0.033) |
| Contracts |  |  |  | -0.0433 |
|  |  |  |  | (0.049) |
| Job Complexity |  |  |  | 1.255^***^ |
|  |  |  |  | (0.041) |
| Relationships at Work |  |  |  | 0.151^***^ |
|  |  |  |  | (0.023) |
| Employee Voice |  |  |  | 0.653^***^ |
|  |  |  |  | (0.040) |
| Health and Wellbeing |  |  |  | 0.704^***^ |
|  |  |  |  | (0.045) |
| Demand and resources |  |  |  | 0.168^***^ |
|  |  |  |  | (0.036) |
| Skills |  |  |  | 0.0255 |
|  |  |  |  | (0.016) |
| Development |  |  |  | 0.677^***^ |
|  |  |  |  | (0.033) |
| Constant | 0.137^***^ | -0.228^***^ | -0.106 | -2.346^***^ |
|  | (0.016) | (0.045) | (0.055) | (0.071) |
| *N* | 13587 | 13587 | 13587 | 13587 |
| *R*^2^ | 0.020 | 0.049 | 0.124 | 0.457 |

Notes: Standard errors in parentheses. Statistical significance * p < 0.05, ** p < 0.01, *** p < 0.001.

Table A-V. Figure II regression results in full (standardised usefulness to organisation)

|  | (1) | (2) | (3) | (4) |
| --- | --- | --- | --- | --- |
| Higher managerial and professional (reference) |  |  |  |  |
|  |  |  |  |  |
| Lower managerial and professional | -0.125^***^ | -0.103^***^ | -0.130^***^ | -0.0183 |
|  | (0.024) | (0.024) | (0.024) | (0.020) |
| Intermediate | -0.293^***^ | -0.250^***^ | -0.244^***^ | -0.00563 |
|  | (0.028) | (0.030) | (0.031) | (0.027) |
| Small employers and own account | -0.0543 | -0.0738 | -0.193^***^ | 0.00888 |
|  | (0.043) | (0.044) | (0.053) | (0.047) |
| Lower supervisory and technical | -0.277^***^ | -0.200^***^ | -0.155^**^ | 0.00667 |
|  | (0.048) | (0.048) | (0.048) | (0.042) |
| Semi-routine | -0.310^***^ | -0.270^***^ | -0.232^***^ | 0.0901^**^ |
|  | (0.037) | (0.037) | (0.038) | (0.033) |
| Routine | -0.356^***^ | -0.291^***^ | -0.253^***^ | 0.126^**^ |
|  | (0.049) | (0.050) | (0.050) | (0.044) |
| Survey year: 2018 (reference) |  |  |  |  |
|  |  |  |  |  |
| Survey year: 2019 |  | 0.0718^**^ | 0.0641^**^ | 0.0814^***^ |
|  |  | (0.023) | (0.023) | (0.020) |
| Survey year: 2020 |  | 0.0290 | 0.0249 | 0.0845^***^ |
|  |  | (0.022) | (0.022) | (0.020) |
| Age: 18-29 (reference) |  |  |  |  |
|  |  |  |  |  |
| Age: 30-39 |  | 0.111^**^ | 0.107^*^ | 0.109^**^ |
|  |  | (0.043) | (0.042) | (0.036) |
| Age: 40-49 |  | 0.183^***^ | 0.154^***^ | 0.180^***^ |
|  |  | (0.041) | (0.041) | (0.036) |
| Age: 50-59 |  | 0.190^***^ | 0.155^***^ | 0.218^***^ |
|  |  | (0.040) | (0.041) | (0.036) |
| Age: 60+ |  | 0.382^***^ | 0.327^***^ | 0.293^***^ |
|  |  | (0.041) | (0.044) | (0.039) |
| Female |  | 0.0844^***^ | 0.0490^*^ | 0.0661^***^ |
|  |  | (0.019) | (0.020) | (0.018) |
| Other than white ethnicity |  | 0.00129 | -0.00417 | -0.0323 |
|  |  | (0.038) | (0.038) | (0.032) |
| Household size: 1 |  |  |  |  |
|  |  |  |  |  |
| Household size: 2 |  | 0.0574^*^ | 0.0604^*^ | 0.0162 |
|  |  | (0.025) | (0.025) | (0.022) |
| Household size: 3 |  | 0.00340 | 0.00862 | -0.0311 |
|  |  | (0.030) | (0.030) | (0.026) |
| Household size: 4 |  | 0.0646^*^ | 0.0712^*^ | -0.00801 |
|  |  | (0.032) | (0.031) | (0.027) |
| Household size: 5 |  | 0.0452 | 0.0517 | -0.0418 |
|  |  | (0.045) | (0.045) | (0.039) |
| Qualifications: Below degree |  |  |  |  |
|  |  |  |  |  |
| Qualifications: Undergraduate |  | 0.122^***^ | 0.0923^***^ | 0.0493^*^ |
|  |  | (0.023) | (0.023) | (0.020) |
| Qualifications: Postgraduate |  | 0.228^***^ | 0.182^***^ | 0.0891^***^ |
|  |  |  |  |  |
| Region: Northern England |  |  |  |  |
|  |  |  |  |  |
| Region: Midlands |  | -0.00797 | -0.00334 | 0.0209 |
|  |  | (0.031) | (0.030) | (0.026) |
| Region: East England |  | -0.0383 | -0.0329 | -0.0243 |
|  |  | (0.038) | (0.038) | (0.032) |
| Region: London |  | -0.00834 | 0.0121 | 0.0131 |
|  |  | (0.034) | (0.034) | (0.029) |
| Region: Southern England |  | 0.0342 | 0.0437 | 0.0336 |
|  |  | (0.025) | (0.025) | (0.022) |
| Region: Wales |  | -0.0491 | -0.0525 | -0.0413 |
|  |  | (0.047) | (0.047) | (0.041) |
| Region: Scotland |  | 0.0452 | 0.0533 | 0.0249 |
|  |  | (0.033) | (0.032) | (0.028) |
| Region: Northern Ireland |  | -0.00168 | 0.00155 | 0.0109 |
|  |  | (0.093) | (0.093) | (0.087) |
| Tenure: <=1 year (reference) |  |  |  |  |
|  |  |  |  |  |
| Tenure: <=5 years |  |  | -0.0120 | 0.0497 |
|  |  |  | (0.033) | (0.028) |
| Tenure: <=15 years |  |  | -0.0434 | 0.0102 |
|  |  |  | (0.032) | (0.028) |
| Tenure: 15+ |  |  | -0.0213 | 0.0257 |
|  |  |  | (0.035) | (0.030) |
| Part-time |  |  | -0.0119 | 0.0336 |
|  |  |  | (0.023) | (0.022) |
| Self-employed |  |  | 0.0315 | -0.0147 |
|  |  |  | (0.040) | (0.040) |
| Temporary contract |  |  | 0.0370 | 0.0487 |
|  |  |  | (0.055) | (0.053) |
| Union |  |  | 0.0500 | -0.0394 |
|  |  |  | (0.026) | (0.024) |
| Workplace size: 1-9 workers (reference) |  |  |  |  |
|  |  |  |  |  |
| Workplace size: 10-249 |  |  | -0.124^***^ | -0.0961^***^ |
|  |  |  | (0.031) | (0.028) |
| Workplace size: 250+ |  |  | -0.256^***^ | -0.239^***^ |
|  |  |  | (0.031) | (0.028) |
| Industry: Industry (reference) |  |  |  |  |
|  |  |  |  |  |
| Industry: Consumer services |  |  | -0.0801^**^ | 0.0105 |
|  |  |  | (0.028) | (0.025) |
| Industry: Business services |  |  | -0.0165 | -0.0440 |
|  |  |  | (0.029) | (0.025) |
| Industry: Public services |  |  | 0.255^***^ | 0.206^***^ |
|  |  |  | (0.030) | (0.027) |
| Work-Life Balance |  |  |  | 0.0947 |
|  |  |  |  | (0.057) |
| Pay and Benefits |  |  |  | 0.189^***^ |
|  |  |  |  | (0.043) |
| Contracts |  |  |  | 0.0695 |
|  |  |  |  | (0.068) |
| Job Complexity |  |  |  | 1.332^***^ |
|  |  |  |  | (0.056) |
| Relationships at Work |  |  |  | 0.251^***^ |
|  |  |  |  | (0.031) |
| Employee Voice |  |  |  | 0.594^***^ |
|  |  |  |  | (0.052) |
| Health and Wellbeing |  |  |  | 0.664^***^ |
|  |  |  |  | (0.061) |
| Demand and resources |  |  |  | 0.262^***^ |
|  |  |  |  | (0.049) |
| Skills |  |  |  | -0.00350 |
|  |  |  |  | (0.021) |
| Development |  |  |  | 0.586^***^ |
|  |  |  |  | (0.044) |
| Constant | 0.186^***^ | -0.246^***^ | -0.0362 | -2.474^***^ |
|  | (0.018) | (0.054) | (0.067) | (0.097) |
| *N* | 13587 | 13587 | 13587 | 13587 |
| *R*^2^ | 0.015 | 0.037 | 0.059 | 0.305 |

Notes: Standard errors in parentheses. Statistical significance * p < 0.05, ** p < 0.01, *** p < 0.001.

Table A-VI. Figure III regression results in full (standardised usefulness to society)

|  | (1) | (2) | (3) | (4) |
| --- | --- | --- | --- | --- |
| Higher managerial and professional (reference) |  |  |  |  |
|  |  |  |  |  |
| Lower managerial and professional | 0.0289 | 0.0552^*^ | -0.0631^**^ | 0.0222 |
|  | (0.025) | (0.025) | (0.024) | (0.021) |
| Intermediate | -0.232^***^ | -0.185^***^ | -0.239^***^ | -0.0373 |
|  | (0.030) | (0.031) | (0.029) | (0.027) |
| Small employers and own account | 0.00442 | 0.0150 | -0.0144 | 0.142^***^ |
|  | (0.039) | (0.039) | (0.046) | (0.042) |
| Lower supervisory and technical | -0.157^***^ | -0.0786 | 0.00159 | 0.123^**^ |
|  | (0.043) | (0.044) | (0.043) | (0.039) |
| Semi-routine | -0.0579 | -0.0147 | -0.0538 | 0.206^***^ |
|  | (0.036) | (0.037) | (0.035) | (0.032) |
| Routine | -0.193^***^ | -0.121^*^ | -0.0706 | 0.248^***^ |
|  | (0.046) | (0.048) | (0.046) | (0.042) |
| Survey year: 2018 (reference) |  |  |  |  |
|  |  |  |  |  |
| Survey year: 2019 |  | 0.0860^***^ | 0.0741^***^ | 0.0759^***^ |
|  |  | (0.023) | (0.021) | (0.020) |
| Survey year: 2020 |  | 0.0307 | 0.0272 | 0.0717^***^ |
|  |  | (0.022) | (0.020) | (0.020) |
| Age: 18-29 (reference) |  |  |  |  |
|  |  |  |  |  |
| Age: 30-39 |  | 0.0586 | 0.0229 | 0.0283 |
|  |  | (0.042) | (0.039) | (0.035) |
| Age: 40-49 |  | 0.138^***^ | 0.0570 | 0.0910^**^ |
|  |  | (0.040) | (0.038) | (0.035) |
| Age: 50-59 |  | 0.155^***^ | 0.0578 | 0.128^***^ |
|  |  | (0.039) | (0.038) | (0.034) |
| Age: 60+ |  | 0.317^***^ | 0.220^***^ | 0.205^***^ |
|  |  | (0.041) | (0.041) | (0.037) |
| Female |  | 0.0979^***^ | 0.0123 | 0.0228 |
|  |  | (0.019) | (0.019) | (0.017) |
| Other than white ethnicity |  | 0.156^***^ | 0.144^***^ | 0.110^**^ |
|  |  | (0.039) | (0.037) | (0.034) |
| Household size: 1 |  |  |  |  |
|  |  |  |  |  |
| Household size: 2 |  | -0.0534^*^ | -0.0249 | -0.0549^*^ |
|  |  | (0.025) | (0.023) | (0.021) |
| Household size: 3 |  | -0.0722^*^ | -0.0544^*^ | -0.0842^***^ |
|  |  | (0.030) | (0.027) | (0.025) |
| Household size: 4 |  | -0.0101 | 0.0181 | -0.0447 |
|  |  | (0.032) | (0.030) | (0.027) |
| Household size: 5 |  | 0.0145 | 0.0420 | -0.0289 |
|  |  | (0.044) | (0.041) | (0.037) |
| Qualifications: Below degree |  |  |  |  |
|  |  |  |  |  |
| Qualifications: Undergraduate |  | 0.0849^***^ | 0.0197 | -0.00246 |
|  |  | (0.023) | (0.022) | (0.019) |
| Qualifications: Postgraduate |  | 0.267^***^ | 0.148^***^ | 0.0781^***^ |
|  |  | (0.024) | (0.022) | (0.020) |
| Region: Northern England |  |  |  |  |
|  |  |  |  |  |
| Region: Midlands |  | -0.0457 | -0.0239 | -0.00583 |
|  |  | (0.031) | (0.028) | (0.025) |
| Region: East England |  | -0.0822^*^ | -0.0470 | -0.0391 |
|  |  | (0.036) | (0.034) | (0.030) |
| Region: London |  | -0.0736^*^ | -0.00222 | 0.00344 |
|  |  | (0.034) | (0.031) | (0.028) |
| Region: Southern England |  | -0.0185 | 0.0303 | 0.0231 |
|  |  | (0.026) | (0.024) | (0.022) |
| Region: Wales |  | -0.0173 | -0.00668 | -0.00526 |
|  |  | (0.045) | (0.042) | (0.037) |
| Region: Scotland |  | 0.0634 | 0.0929^**^ | 0.0655^*^ |
|  |  | (0.034) | (0.031) | (0.028) |
| Region: Northern Ireland |  | -0.0304 | -0.0128 | -0.00771 |
|  |  | (0.094) | (0.086) | (0.081) |
| Tenure: <=1 year (reference) |  |  |  |  |
|  |  |  |  |  |
| Tenure: <=5 years |  |  | -0.0211 | 0.0252 |
|  |  |  | (0.032) | (0.028) |
| Tenure: <=15 years |  |  | -0.0832^**^ | -0.0384 |
|  |  |  | (0.031) | (0.028) |
| Tenure: 15+ |  |  | -0.0622 | -0.0271 |
|  |  |  | (0.034) | (0.030) |
| Part-time |  |  | 0.0116 | 0.0669^**^ |
|  |  |  | (0.021) | (0.021) |
| Self-employed |  |  | 0.168^***^ | 0.169^***^ |
|  |  |  | (0.037) | (0.039) |
| Temporary contract |  |  | 0.0175 | 0.0115 |
|  |  |  | (0.056) | (0.052) |
| Union |  |  | 0.194^***^ | 0.124^***^ |
|  |  |  | (0.024) | (0.024) |
| Workplace size: 1-9 workers (reference) |  |  |  |  |
|  |  |  |  |  |
| Workplace size: 10-249 |  |  | -0.0228 | -0.0164 |
|  |  |  | (0.031) | (0.028) |
| Workplace size: 250+ |  |  | -0.0226 | -0.0270 |
|  |  |  | (0.030) | (0.028) |
| Industry: Industry (reference) |  |  |  |  |
|  |  |  |  |  |
| Industry: Consumer services |  |  | 0.0126 | 0.0815^***^ |
|  |  |  | (0.027) | (0.024) |
| Industry: Business services |  |  | -0.0258 | -0.0556^*^ |
|  |  |  | (0.028) | (0.025) |
| Industry: Public services |  |  | 0.842^***^ | 0.773^***^ |
|  |  |  | (0.029) | (0.026) |
| Work-Life Balance |  |  |  | 0.0961 |
|  |  |  |  | (0.054) |
| Pay and Benefits |  |  |  | -0.0142 |
|  |  |  |  | (0.041) |
| Contracts |  |  |  | -0.00767 |
|  |  |  |  | (0.061) |
| Job Complexity |  |  |  | 1.209^***^ |
|  |  |  |  | (0.051) |
| Relationships at Work |  |  |  | -0.0104 |
|  |  |  |  | (0.019) |
| Employee Voice |  |  |  | 0.453^***^ |
|  |  |  |  | (0.052) |
| Health and Wellbeing |  |  |  | 0.679^***^ |
|  |  |  |  | (0.057) |
| Demand and resources |  |  |  | 0.0595 |
|  |  |  |  | (0.042) |
| Skills |  |  |  | 0.0704^***^ |
|  |  |  |  | (0.020) |
| Development |  |  |  | 0.610^***^ |
|  |  |  |  | (0.041) |
| Constant | 0.0533^**^ | -0.261^***^ | -0.298^***^ | -2.154^***^ |
|  | (0.019) | (0.053) | (0.064) | (0.089) |
| *N* | 13587 | 13587 | 13587 | 13587 |
| *R*^2^ | 0.010 | 0.035 | 0.174 | 0.337 |

Notes: Standard errors in parentheses. Statistical significance * p < 0.05, ** p < 0.01, *** p < 0.001.

Table A-VII. Figure IV regression results in full (standardised organisation’s purpose)

|  | (1) | (2) | (3) | (4) |
| --- | --- | --- | --- | --- |
| Higher managerial and professional (reference) |  |  |  |  |
|  |  |  |  |  |
| Lower managerial and professional | -0.0959^***^ | -0.0760^**^ | -0.128^***^ | -0.000 |
|  | (0.025) | (0.025) | (0.025) | (0.021) |
| Intermediate | -0.294^***^ | -0.251^***^ | -0.254^***^ | 0.0186 |
|  | (0.029) | (0.031) | (0.031) | (0.026) |
| Small employers and own account | 0.101^*^ | 0.114^**^ | -0.143^**^ | 0.103^*^ |
|  | (0.043) | (0.044) | (0.052) | (0.046) |
| Lower supervisory and technical | -0.329^***^ | -0.237^***^ | -0.188^***^ | -0.0102 |
|  | (0.047) | (0.048) | (0.048) | (0.039) |
| Semi-routine | -0.339^***^ | -0.284^***^ | -0.273^***^ | 0.0728^*^ |
|  | (0.037) | (0.038) | (0.039) | (0.031) |
| Routine | -0.464^***^ | -0.367^***^ | -0.346^***^ | 0.0721 |
|  | (0.045) | (0.047) | (0.048) | (0.040) |
| Survey year: 2018 (reference) |  |  |  |  |
|  |  |  |  |  |
| Survey year: 2019 |  | 0.0401 | 0.0236 | 0.0292 |
|  |  | (0.023) | (0.023) | (0.019) |
| Survey year: 2020 |  | -0.000128 | -0.0104 | 0.0427^*^ |
|  |  | (0.023) | (0.022) | (0.019) |
| Age: 18-29 (reference) |  |  |  |  |
|  |  |  |  |  |
| Age: 30-39 |  | 0.0863^*^ | 0.0970^*^ | 0.106^**^ |
|  |  | (0.042) | (0.041) | (0.033) |
| Age: 40-49 |  | 0.127^**^ | 0.114^**^ | 0.158^***^ |
|  |  | (0.041) | (0.040) | (0.033) |
| Age: 50-59 |  | 0.122^**^ | 0.104^**^ | 0.199^***^ |
|  |  | (0.040) | (0.040) | (0.033) |
| Age: 60+ |  | 0.288^***^ | 0.252^***^ | 0.235^***^ |
|  |  | (0.042) | (0.043) | (0.035) |
| Female |  | 0.114^***^ | 0.0653^**^ | 0.0902^***^ |
|  |  | (0.020) | (0.020) | (0.017) |
| Other than white ethnicity |  | -0.0221 | -0.0318 | -0.0724^*^ |
|  |  | (0.041) | (0.040) | (0.032) |
| Household size: 1 |  |  |  |  |
|  |  |  |  |  |
| Household size: 2 |  | 0.0347 | 0.0449 | -0.00132 |
|  |  | (0.026) | (0.025) | (0.021) |
| Household size: 3 |  | 0.00769 | 0.0218 | -0.0261 |
|  |  | (0.030) | (0.030) | (0.024) |
| Household size: 4 |  | 0.0781^*^ | 0.0968^**^ | 0.00217 |
|  |  | (0.032) | (0.031) | (0.025) |
| Household size: 5 |  | 0.0985^*^ | 0.112^*^ | -0.00237 |
|  |  | (0.045) | (0.044) | (0.035) |
| Qualifications: Below degree |  |  |  |  |
|  |  |  |  |  |
| Qualifications: Undergraduate |  | 0.159^***^ | 0.120^***^ | 0.0793^***^ |
|  |  | (0.024) | (0.024) | (0.019) |
| Qualifications: Postgraduate |  | 0.260^***^ | 0.197^***^ | 0.102^***^ |
|  |  | (0.024) | (0.024) | (0.020) |
| Region: Northern England |  |  |  |  |
|  |  |  |  |  |
| Region: Midlands |  | -0.0563 | -0.0497 | -0.0237 |
|  |  | (0.031) | (0.030) | (0.025) |
| Region: East England |  | -0.0539 | -0.0431 | -0.0303 |
|  |  | (0.036) | (0.036) | (0.029) |
| Region: London |  | 0.0139 | 0.0405 | 0.0320 |
|  |  | (0.034) | (0.034) | (0.028) |
| Region: Southern England |  | -0.00139 | 0.0125 | -0.000393 |
|  |  | (0.026) | (0.026) | (0.021) |
| Region: Wales |  | -0.0829 | -0.0821 | -0.0761^*^ |
|  |  | (0.045) | (0.044) | (0.036) |
| Region: Scotland |  | 0.0506 | 0.0659^*^ | 0.0311 |
|  |  | (0.033) | (0.032) | (0.027) |
| Region: Northern Ireland |  | -0.0857 | -0.0686 | -0.0666 |
|  |  | (0.103) | (0.103) | (0.068) |
| Tenure: <=1 year (reference) |  |  |  |  |
|  |  |  |  |  |
| Tenure: <=5 years |  |  | -0.0929^**^ | -0.0164 |
|  |  |  | (0.033) | (0.026) |
| Tenure: <=15 years |  |  | -0.152^***^ | -0.0737^**^ |
|  |  |  | (0.033) | (0.026) |
| Tenure: 15+ |  |  | -0.161^***^ | -0.0854^**^ |
|  |  |  | (0.035) | (0.028) |
| Part-time |  |  | -0.0278 | 0.0114 |
|  |  |  | (0.023) | (0.020) |
| Self-employed |  |  | 0.245^***^ | 0.261^***^ |
|  |  |  | (0.041) | (0.040) |
| Temporary contract |  |  | 0.0363 | -0.00459 |
|  |  |  | (0.061) | (0.053) |
| Union |  |  | 0.0585^*^ | -0.0810^***^ |
|  |  |  | (0.027) | (0.024) |
| Workplace size: 1-9 workers (reference) |  |  |  |  |
|  |  |  |  |  |
| Workplace size: 10-249 |  |  | -0.0816^*^ | -0.0676^*^ |
|  |  |  | (0.033) | (0.026) |
| Workplace size: 250+ |  |  | -0.191^***^ | -0.206^***^ |
|  |  |  | (0.032) | (0.026) |
| Industry: Industry (reference) |  |  |  |  |
|  |  |  |  |  |
| Industry: Consumer services |  |  | 0.00808 | 0.103^***^ |
|  |  |  | (0.028) | (0.022) |
| Industry: Business services |  |  | -0.0126 | -0.0442 |
|  |  |  | (0.029) | (0.023) |
| Industry: Public services |  |  | 0.416^***^ | 0.365^***^ |
|  |  |  | (0.031) | (0.025) |
| Work-Life Balance |  |  |  | 0.0690 |
|  |  |  |  | (0.052) |
| Pay and Benefits |  |  |  | 0.294^***^ |
|  |  |  |  | (0.040) |
| Contracts |  |  |  | -0.183^**^ |
|  |  |  |  | (0.062) |
| Job Complexity |  |  |  | 1.216^***^ |
|  |  |  |  | (0.051) |
| Relationships at Work |  |  |  | 0.163^***^ |
|  |  |  |  | (0.028) |
| Employee Voice |  |  |  | 0.903^***^ |
|  |  |  |  | (0.051) |
| Health and Wellbeing |  |  |  | 0.799^***^ |
|  |  |  |  | (0.057) |
| Demand and resources |  |  |  | 0.176^***^ |
|  |  |  |  | (0.043) |
| Skills |  |  |  | 0.0176 |
|  |  |  |  | (0.020) |
| Development |  |  |  | 0.870^***^ |
|  |  |  |  | (0.043) |
| Constant | 0.171^***^ | -0.199^***^ | -0.0192 | -2.435^***^ |
|  | (0.019) | (0.054) | (0.068) | (0.088) |
| *N* | 13587 | 13587 | 13587 | 13587 |
| *R*^2^ | 0.024 | 0.044 | 0.084 | 0.408 |

Notes: Standard errors in parentheses. Statistical significance * p < 0.05, ** p < 0.01, *** p < 0.001.

Table A-VIII. Decomposition of the mediating role of job quality on occupational class and overall meaningfulness index (contribution of each variable to change in coefficients in Model 3 vs. Model 4 in Table A-IV)

|  | LMP | I | SE&OA | LS&T | S-R | R |
| --- | --- | --- | --- | --- | --- | --- |
| Pay and benefits | -0.008 | -0.010 | -0.009 | -0.011 | -0.015 | -0.012 |
| Contracts | 0.000 | -0.000 | -0.002 | 0.002 | 0.001 | 0.002 |
| Demands-resources | -0.005 | -0.004 | -0.004 | -0.006 | -0.011 | -0.009 |
| Skills | -0.001 | -0.003 | -0.002 | -0.002 | -0.004 | -0.005 |
| Development | -0.028 | -0.068 | -0.044 | -0.041 | -0.062 | -0.097 |
| Job complexity | -0.044 | -0.119 | -0.083 | -0.061 | -0.161 | -0.188 |
| Work life balance | -0.001 | 0.001 | -0.001 | 0.001 | -0.000 | 0.001 |
| Relationships | -0.003 | -0.007 | -0.009 | -0.008 | -0.009 | -0.010 |
| Voice | -0.011 | -0.024 | -0.043 | -0.022 | -0.033 | -0.044 |
| Health and wellbeing | -0.007 | -0.000 | -0.008 | -0.007 | -0.013 | -0.008 |
| **Total gap** | **-0.108** | **-0.234** | **-0.205** | **-0.155** | **-0.307** | **-0.370** |

Notes: Decomposition of change in effect size of each class category relative to higher managerial and professional occupations due to the inclusion of each job quality variable conditional on the controls i.e., Model 3 versus Model 4 in Table A-IV. LMP are lower managerial and professional occupations, I are intermediate occupations, SE&OA are small employers and own account workers, LS&T are lower supervisory and technical occupations, S-R are semi-routine occupations, and R are routine occupations.

Table A-IX. Decomposition of the mediating role of job quality on occupational class and usefulness to organisation (contribution of each variable to change in coefficients in Model 3 vs. Model 4 in Table A-V)

|  | LMP | I | SE&OA | LS&T | S-R | R |
| --- | --- | --- | --- | --- | --- | --- |
| Pay and benefits | -0.010 | -0.013 | -0.012 | -0.014 | -0.020 | -0.016 |
| Contracts | -0.000 | 0.000 | 0.003 | -0.003 | -0.002 | -0.003 |
| Demands-resources | -0.007 | -0.007 | -0.006 | -0.009 | -0.017 | -0.014 |
| Skills | 0.000 | 0.001 | 0.000 | 0.001 | 0.001 | 0.001 |
| Development | -0.024 | -0.058 | -0.037 | -0.035 | -0.053 | -0.082 |
| Job complexity | -0.047 | -0.127 | -0.089 | -0.065 | -0.171 | -0.200 |
| Work life balance | -0.001 | 0.002 | -0.001 | 0.001 | -0.000 | 0.001 |
| Relationships | -0.005 | -0.012 | -0.016 | -0.013 | -0.015 | -0.017 |
| Voice | -0.010 | -0.022 | -0.040 | -0.020 | -0.031 | -0.040 |
| Health and wellbeing | -0.007 | -0.000 | -0.007 | -0.006 | -0.012 | -0.007 |
| **Total gap** | **-0.111** | **-0.236** | **-0.205** | **-0.163** | **-0.320** | **-0.377** |

Notes: Decomposition of change in effect size of each class category relative to higher managerial and professional occupations due to the inclusion of each job quality variable conditional on the controls i.e., Model 3 versus Model 4 in Table A-V. LMP are lower managerial and professional occupations, I are intermediate occupations, SE&OA are small employers and own account workers, LS&T are lower supervisory and technical occupations, S-R are semi-routine occupations, and R are routine occupations.

Table A-X. Decomposition of the mediating role of job quality on occupational class and usefulness to society (contribution of each variable to change in coefficients in Model 3 vs. Model 4 in Table A-VI)

|  | LMP | I | SE&OA | LS&T | S-R | R |
| --- | --- | --- | --- | --- | --- | --- |
| Pay and benefits | 0.002 | 0.002 | 0.002 | 0.002 | 0.003 | 0.003 |
| Contracts | 0.000 | -0.000 | -0.001 | 0.001 | 0.000 | 0.001 |
| Demands-resources | -0.002 | -0.002 | -0.001 | -0.002 | -0.004 | -0.003 |
| Skills | -0.004 | -0.009 | -0.005 | -0.006 | -0.010 | -0.012 |
| Development | -0.025 | -0.060 | -0.039 | -0.036 | -0.055 | -0.085 |
| Job complexity | -0.043 | -0.116 | -0.081 | -0.059 | -0.156 | -0.182 |
| Work life balance | -0.001 | 0.002 | -0.001 | 0.001 | -0.000 | 0.001 |
| Relationships | -0.001 | -0.002 | -0.002 | -0.002 | -0.002 | -0.002 |
| Voice | -0.008 | -0.017 | -0.031 | -0.015 | -0.024 | -0.031 |
| Health and wellbeing | -0.007 | -0.000 | -0.007 | -0.006 | -0.012 | -0.007 |
| **Total gap** | **-0.089** | **-0.202** | **-0.166** | **-0.122** | **-0.260** | **-0.317** |

Notes: Decomposition of change in effect size of each class category relative to higher managerial and professional occupations due to the inclusion of each job quality variable conditional on the controls i.e., Model 3 versus Model 4 in Table A-VI. LMP are lower managerial and professional occupations, I are intermediate occupations, SE&OA are small employers and own account workers, LS&T are lower supervisory and technical occupations, S-R are semi-routine occupations, and R are routine occupations.

Table A-XI. Decomposition of the mediating role of job quality on occupational class and organisation’s purpose (contribution of each variable to change in coefficients in Model 3 vs. Model 4 in Table A-VII)

|  | LMP | I | SE&OA | LS&T | S-R | R |
| --- | --- | --- | --- | --- | --- | --- |
| Pay and benefits | -0.015 | -0.020 | -0.018 | -0.021 | -0.030 | -0.024 |
| Contracts | 0.001 | -0.000 | -0.007 | 0.007 | 0.006 | 0.007 |
| Demands-resources | -0.005 | -0.005 | -0.004 | -0.006 | -0.012 | -0.010 |
| Skills | -0.001 | -0.002 | -0.001 | -0.002 | -0.003 | -0.003 |
| Development | -0.036 | -0.087 | -0.056 | -0.052 | -0.079 | -0.123 |
| Job complexity | -0.043 | -0.115 | -0.080 | -0.059 | -0.155 | -0.181 |
| Work life balance | -0.000 | 0.001 | -0.000 | 0.001 | -0.000 | 0.000 |
| Relationships | -0.003 | -0.008 | -0.010 | -0.009 | -0.009 | -0.011 |
| Voice | -0.016 | -0.032 | -0.060 | -0.030 | -0.046 | -0.061 |
| Health and wellbeing | -0.008 | -0.000 | -0.009 | -0.007 | -0.015 | -0.009 |
| **Total gap** | **-0.126** | **-0.268** | **-0.245** | **-0.178** | **-0.343** | **-0.415** |

Notes: Decomposition of change in effect size of each class category relative to higher managerial and professional occupations due to the inclusion of each job quality variable conditional on the controls i.e., Model 3 versus Model 4 in Table A-VII. LMP are lower managerial and professional occupations, I are intermediate occupations, SE&OA are small employers and own account workers, LS&T are lower supervisory and technical occupations, S-R are semi-routine occupations, and R are routine occupations.

Figure A-I. Mean meaningfulness index by detailed occupation across specifications


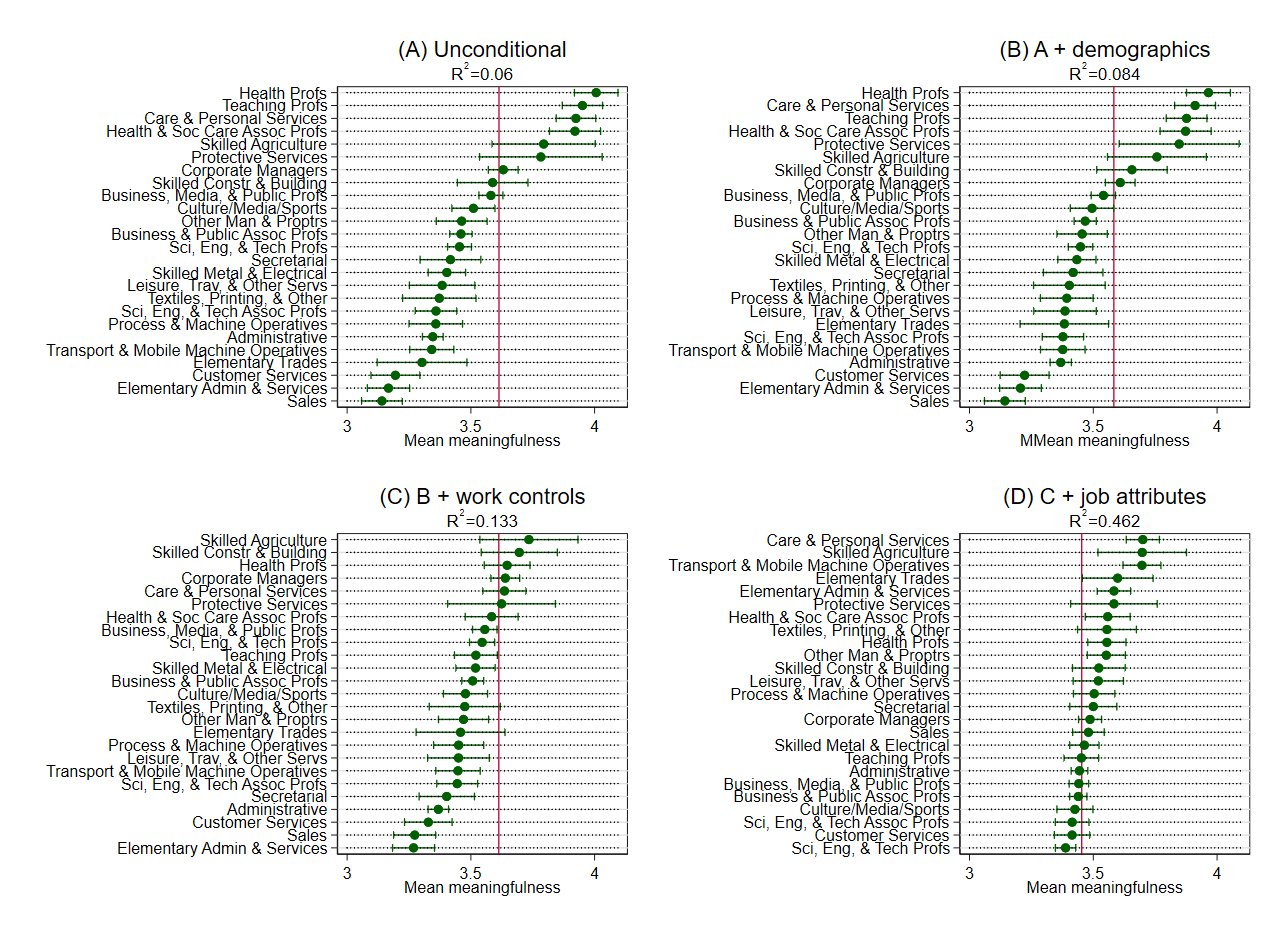


Notes: Results obtained from OLS models with the overall meaningfulness index as the dependent variable and SOC 2-digit categories as the main independent. Dots are means or adjusted means and the horizontal lines are 95% confidence intervals around these estimates. The vertical line is the mean or adjusted mean for higher managerial and professional occupations and can serve as a reference point. See text for controls.

Figure A-II. Mean usefulness to organisation by detailed occupation across specifications


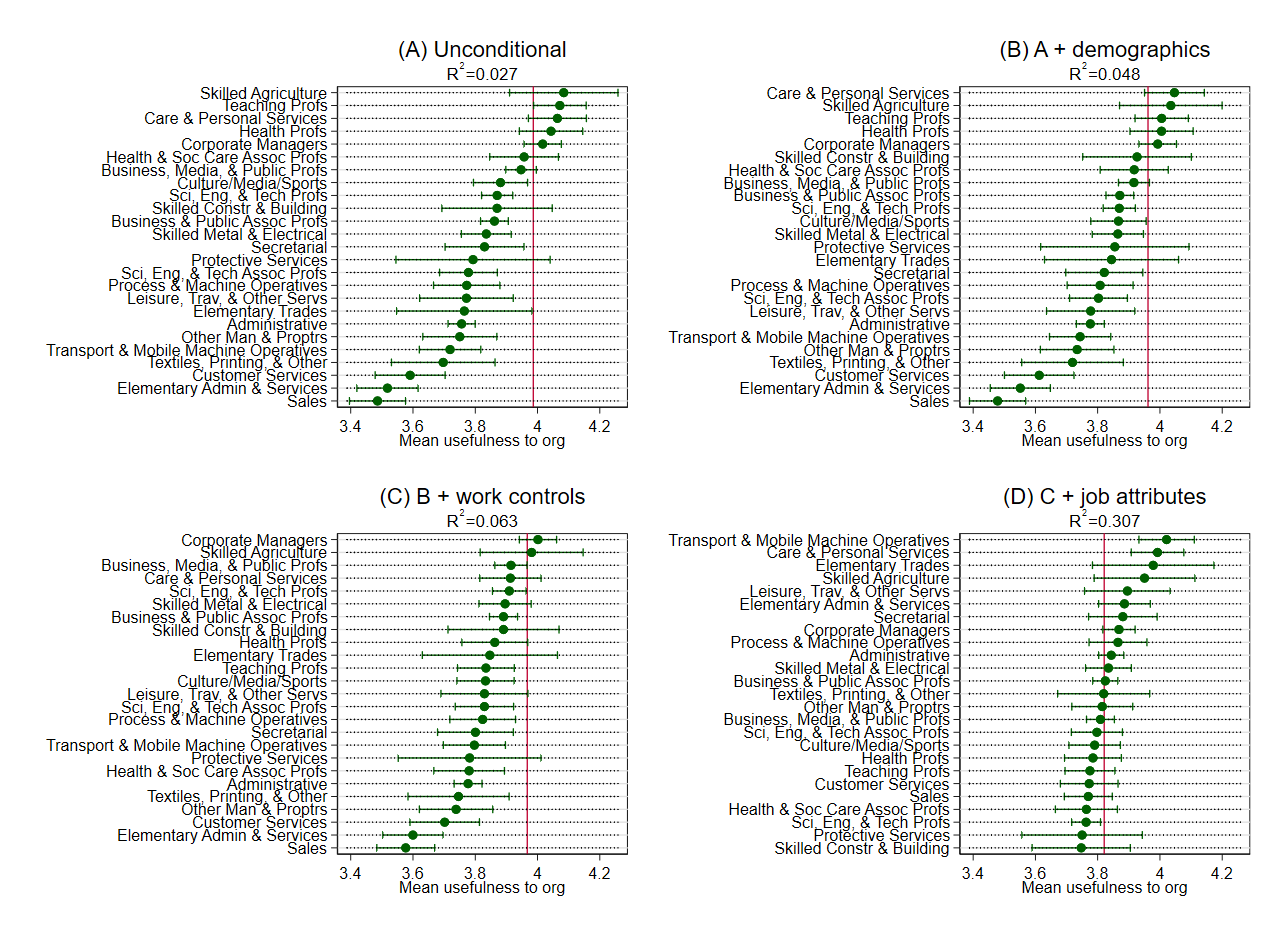
Notes: Results obtained from OLS models with usefulness to organisation as the dependent variable and SOC 2-digit categories as the main independent. Dots are means or adjusted means and the horizontal lines are 95% confidence intervals around these estimates. The vertical line is the mean or adjusted mean for higher managerial and professional occupations and can serve as a reference point. See text for controls.

Figure A-III. Mean usefulness to society by detailed occupation across specifications


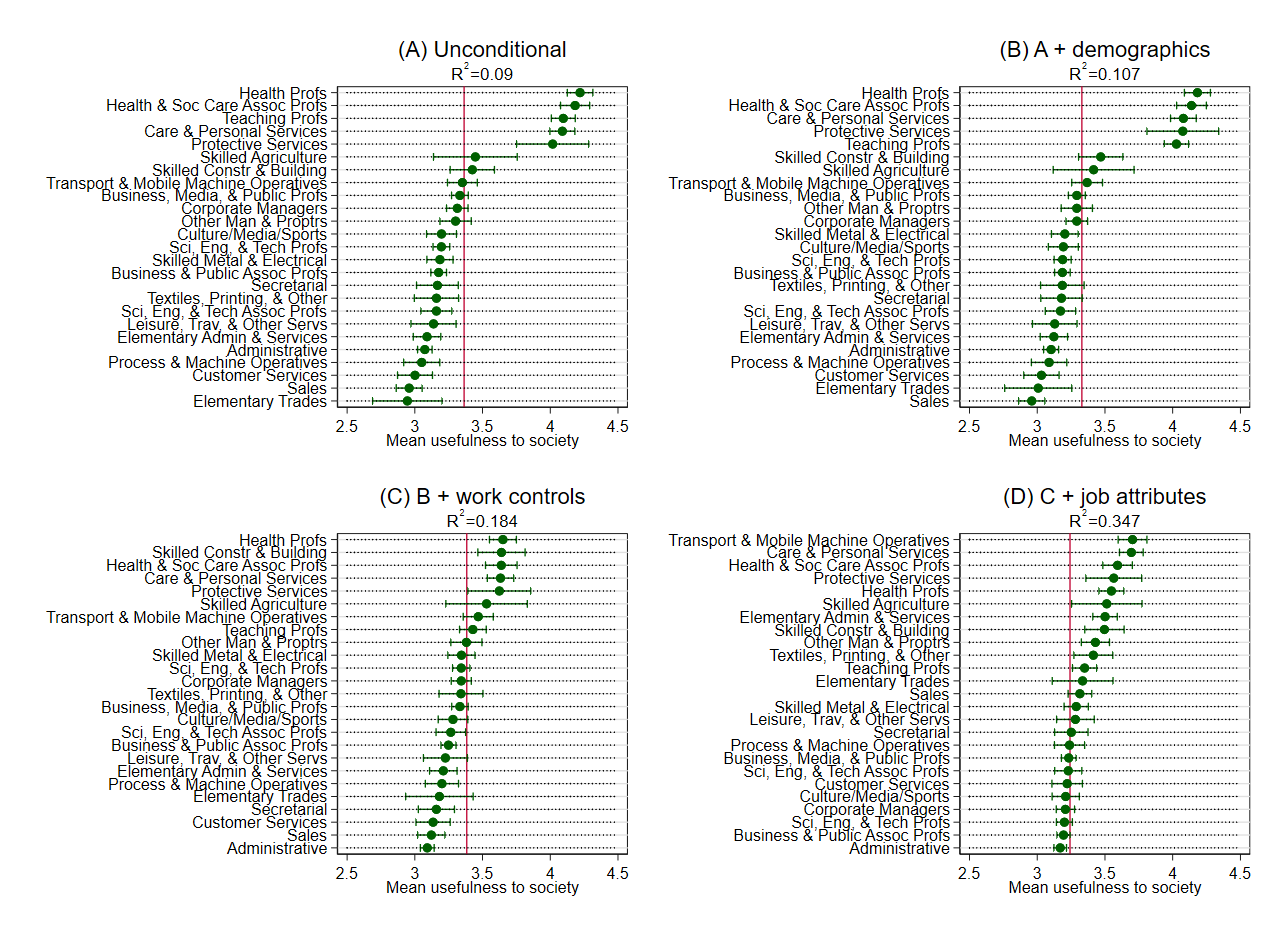


Notes: Results obtained from OLS models with usefulness to society as the dependent variable and SOC 2-digit categories as the main independent. Dots are means or adjusted means and the horizontal lines are 95% confidence intervals around these estimates. The vertical line is the mean or adjusted mean for higher managerial and professional occupations and can serve as a reference point. See text for controls.

Figure A-IV. Mean organisation’s purpose by detailed occupation across specifications


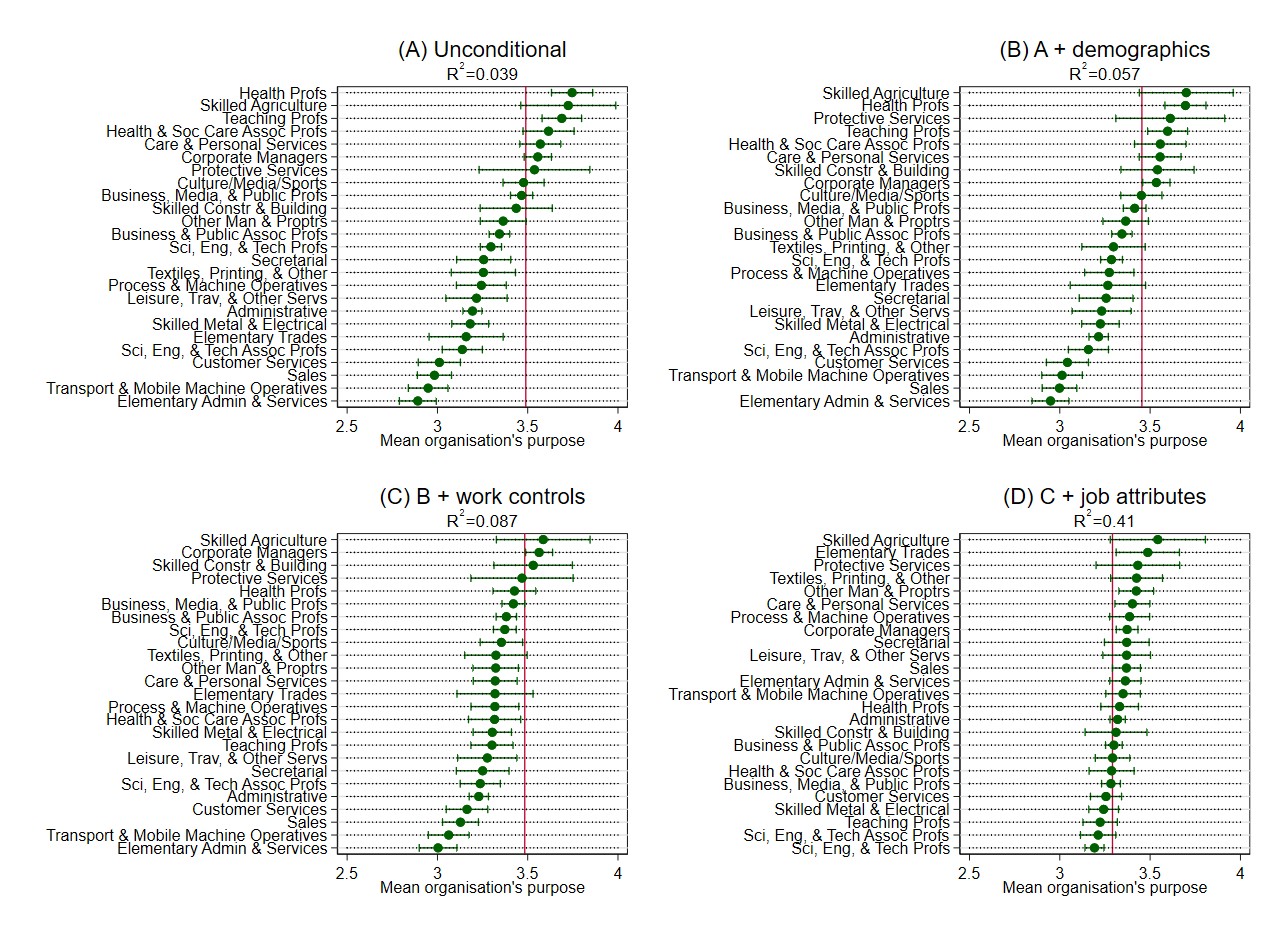


Notes: Results obtained from OLS models with organisation’s purpose as the dependent variable and SOC 2-digit categories as the main independent. Dots are means or adjusted means and the horizontal lines are 95% confidence intervals around these estimates. The vertical line is the mean or adjusted mean for higher managerial and professional occupations and can serve as a reference point. See text for controls.

**References**

**CIPD** 2020. *The CIPD Good Work Index 2020: UK Working Lives Survey Report.* London: Chartered Institute of Personnel and Development (CIPD).
